# Deciphering Aggregation-Induced Tunable Circular Dichroism of AuBP@Ag and AuBP@Au Chiral Core–Shell Plasmonic Nanoparticles

**DOI:** 10.1021/acs.jpclett.6c02669

**Published:** 2026-10-15

**Authors:** Marco Romanelli, Pablo Mariani, Antonio Carone, Anthony Désert, Stephane Parola, Stefano Corni, Ivan Rivalta

**Affiliations:** † Department of Chemical Sciences, University of Padova, via Marzolo 1, 35131 Padova, Italy; ‡ ENS de Lyon, CNRS, Lyon 1 Université, Laboratoire de Chimie, UMR 5182, 46 Allée d’Italie, 69364 Lyon, France; ¶ Sustainable and Bio-inspired Materials, Max Planck Institute of Colloids and Interfaces, Science Park Golm, 14476 Potsdam, Germany; § CNR Institute of Nanoscience, via Campi 213/A, 41125 Modena, Italy; ∥ Padua Quantum Technologies Research Center, University of Padova, 35131 Padova, Italy; ⊥ Department of Industrial Chemistry “Toso Montanari”, University of Bologna, Viale del Risorgimento 4, 40136, Bologna, Italy; # Center for Chemical Catalysis − C3, University of Bologna, via Piero Gobetti 85, 40129 Bologna, Italy

## Abstract

Plasmonic nanoparticles exhibiting strong and tunable chiroptical signals have been drawing much interest recently due to their potential vast applications. A few years ago, it was shown that seed-mediated metal growth can be harnessed to make single nanoparticles intrinsically chiral. Such structures typically manifest strong circular dichroism spectral features that can be further tuned by pure colloidal aggregation, as shown by recent experiments on chiral AuBP@Au and AuBP@Ag core–shell bipyramids. Nevertheless, the origin of such effects, either CD suppression or the rise of new CD bands upon aggregation, has remained obscure. Here, by combining theoretical modeling and experiments, we shed light on such phenomenon, disclosing that single-particle chirality drives the formation of either chiral or achiral aggregates depending on the shape and metal composition of the outer shell, thereby suggesting further avenues to tailor chirality at the nanoscale.

## Graphical abstract

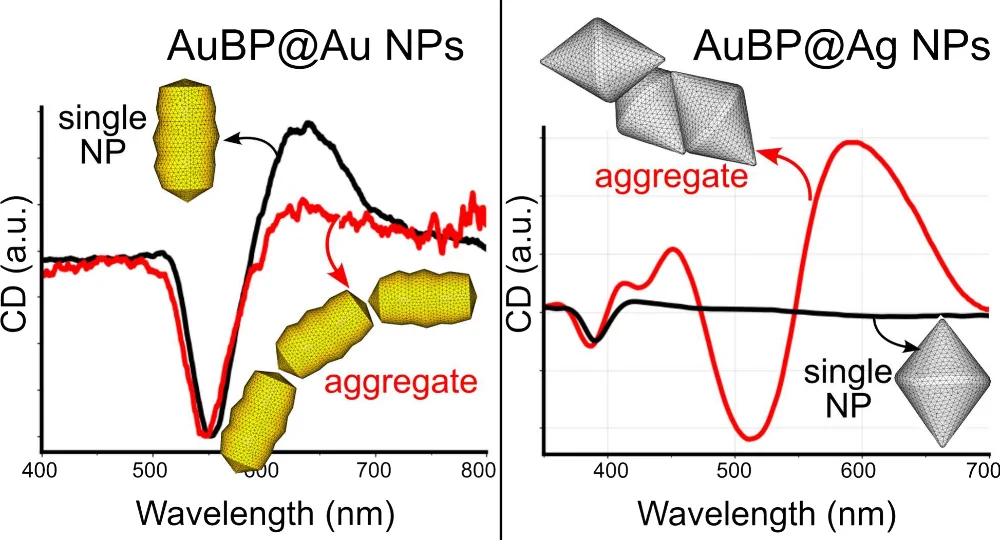

Metallic nanoparticles (NPs) made of plasmonic metals like silver or gold are well-known to support free electron charge oscillations upon light irradiation, named localized surface plasmon resonances (LSPRs).^1,2^ Such a phenomenon typically results in light–matter large interaction cross sections, which make plasmonic NPs extremely bright, leading to strong optical signals.^3^ Recently, there has been rising scientific interest in making such plasmonic NPs chiral, so that by harnessing plasmonic features objects exhibiting strong circular dichroism (CD) can be obtained. This field, known as chiral plasmonics,^4–10^ is nowadays well-established, and the list of possible applications for such structures is extremely vast, ranging from enantioselective catalysis^11^ to biosensing and bioimaging,^6,12,13^ just to name a few. Indeed, the CD signals that can be reached with these structures can be orders of magnitude stronger than with chiral molecules alone,^4^ thereby making chiral plasmonic NPs a promising platform to boost chirality at the nanoscale. Additionally, the NPs’ plasmonic response can be widely tuned to the entire UV–vis–NIR region, for instance by changing the size, shape, embedding environment, and chemical composition of single particles.^14–16^ Even coupling different structures together can result in nontrivial collective effects, which considerably modifies the plasmonic response,^17–22^ thereby constituting an additional control knob that can be harnessed to shape plasmon-mediated chirality at the nanoscale.

In recent years, different strategies have been proposed to obtain plasmonic systems exhibiting strong CD signals. Most of them either combine chiral ligands with plasmonic achiral particles^13,23^ or make use of chiral 3D templates where achiral particles are spatially arranged so that the resulting complex structure features a specific handedness.^24,25^ On the other hand, since 2018 another approach has been introduced by Nam and collaborators,^26^ which results in single particles featuring intrinsic chirality due to the addition of chiral molecules during the synthetic growth of seeds. This strategy has been extended to other systems by several teams, such as Liz-Marzán et al. from Au nanorod seeds^27^ or our team from Au nanobipyramid seeds (AuBPs).^28^ In our case, the growth of AuBPs in the presence of cysteine led to two kinds of chiral core–shell NPs with a Au core and either a Au (AuBP@Au) or Ag (AuBP@Ag) shell. Such particles can be seen as chiral building blocks that can be potentially combined together to further modify chirality at the nanoscale. Indeed, a recent work of some of us^29^ has shown that such chiral core–shell NPs can lead to unexpected chiral effects upon aggregation, resulting in either suppression or appearance of new CD bands that are absent in the CD spectrum of the corresponding single particles, thereby suggesting further viable strategies to shape plasmon-mediated circular dichroism (PCD). Such striking effects are induced by controlled aggregation that is achieved by lowering the surfactant concentration only; that is, no external additional species is needed to promote such aggregation-induced CD modifications.

Recently, several studies^30–35^ have highlighted the possibility of modulating the CD response of chiral plasmonic NPs through control of the aggregation patterns of chiral building blocks. In most cases, however, aggregation is directed by additional chemical linkers or template-assisted approaches that, together with the intrinsic chirality of the individual units, favor specific assembly pathways and aggregate morphologies. For example, Chow et al.^30^ demonstrated that template-assisted assembly of chiral Au nanorods leads to the formation of superlattices containing extended one-dimensional arrays, giving rise to new CD spectral features that depend strongly on the aggregation geometry. Similarly, Zhang et al.^31^ investigated the aggregation of chiral Au nanorods induced by achiral anionic species and observed the spontaneous formation of chiral helical superstructures. Notably, the resulting aggregates displayed CD signals with opposite sign relative to those of the isolated monomers, an effect attributed to the interplay between the geometrical chirality of the nanorods and self-matching during assembly. In a different approach, Trazo et al.^35^ employed depletion-induced self-assembly mediated by surfactant micelles acting as effective depletants to promote aggregation of Au nanorods. Aggregate formation was accompanied by the emergence of new low-energy CD features, while the process remained reversible through modulation of the surfactant concentration. Overall, these studies mainly focused on Au-based nanoparticles and on aggregation processes driven by external directing agents. By contrast, aggregation induced solely by dilution, which is the central phenomenon investigated in our work both for Au- and Ag-based NPs, has remained largely unexplored. Our goal is therefore to elucidate how nanoparticle–nanoparticle interactions and assembly pathways govern the emergence, enhancement, or suppression of CD responses in solution without the explicit use of chiral linkers. These insights provide a foundation for the rational design of dynamic chiroptical systems with potential applications in chiral sensing and enantioselective detection.

In ref 29, TEM images of the aggregated samples showed that in the AuBP@Ag case preferential face-to-face stacking of adjacent particles is often observed, whereas in the case of AuBP@Au the majority of aggregates are composed of linear chains of AuBP@Au NPs with preferential tip-to-tip contact between adjacent particles. Furthermore, in the case of AuBP@Ag, aggregation results in the rise of new red-shifted CD bands that are absent in the single-particle CD spectra, whereas in the AuBP@Au case strong aggregation surprisingly led to suppression of the CD signal, despite the chirality of single AuBP@Au NPs composing the aggregates.^29^ Additionally, it was observed that the CD response of the aggregates depends on the handedness of the constituent chiral NPs, further highlighting the role of the individual nanoparticle chirality in determining the optical response of the resulting assemblies. The origin of such different behavior observed in the experiments was not explored in depth. In this work, by synergistically combining computational modeling and experiments, we shed light on such aggregation-induced PCD effects. Our analysis reveals that in the case of AuBP@Ag aggregates the appearance of new red-shifted CD bands is due to the formation of aggregates with preferential handedness featuring a relative twisting among adjacent NPs. The twisting proved to be fundamental to explaining the CD features of the AuBP@Ag aggregates. On the contrary, in the case of AuBP@Au structures, a different aggregation pattern, featuring mostly a tip-to-tip contact as observed by TEM imaging, leads to aggregates with no preferential handedness, thereby explaining the progressive suppression of CD bands as aggregation is more and more promoted. In this case, extreme aggregation conditions, as investigated in previous experiments by means of strong centrifugation,^29^ can eventually lead to the complete suppression of AuBP@Au CD signals. Our findings pinpoint fundamental differences in the interactions between chiral plasmonic NPs when building blocks of different shapes and metal compositions are considered.

In this work, we focus on two different batches of AuBP@Ag and AuBP@Au NPs that were recently investigated experimentally, namely, AuBP@Ag-147 and AuBP@Au-96.^29^ Statistical analysis of the experimental samples (ref 29) showed that AuBP@Ag-147 feature NPs with an average length of 147 ± 10 nm and an aspect ratio (A.R.) of 1.2, whereas for AuBP@Au-96 NPs the average length is 96 ± 6 nm with an A.R. of 1.8. Based on these mean values, we built two representative computational models (Figure 1) of the single NPs to perform boundary element method (BEM) classical electrodynamics simulations via the MNPBEM Matlab toolbox.^36^ Following previous works,^28,37^ the NP models consist of biconical shapes with spherical caps for both the inner (Au) and outer (Ag or Au) surface meshes as they extensively proved to be effective in describing the optical response of such chiral pentatwinned particles synthesized by seed-mediated growth.^26,28^ As shown in Figure S1, first achiral NPs of dimensions matching the experimental mean values were created; then a slight asymmetrical deformation was applied to both AuBP@Ag and AuBP@Au models to make the structures intrinsically chiral. Further details on the models’ construction can be found in SI Section 1. A direct comparison between the simulated extinction (Figure 1c,d) and CD (Figure 1e,f) spectra with corresponding experimental data of ref 29 validates the models. Indeed, most of the spectral features observed in the experiments are correctly captured by the simulations, thus making our AuBP@Ag and AuBP@Au monomer models a suitable starting point to computationally investigate the role of aggregation on CD, which is the main target of this work.

**Figure 1:**
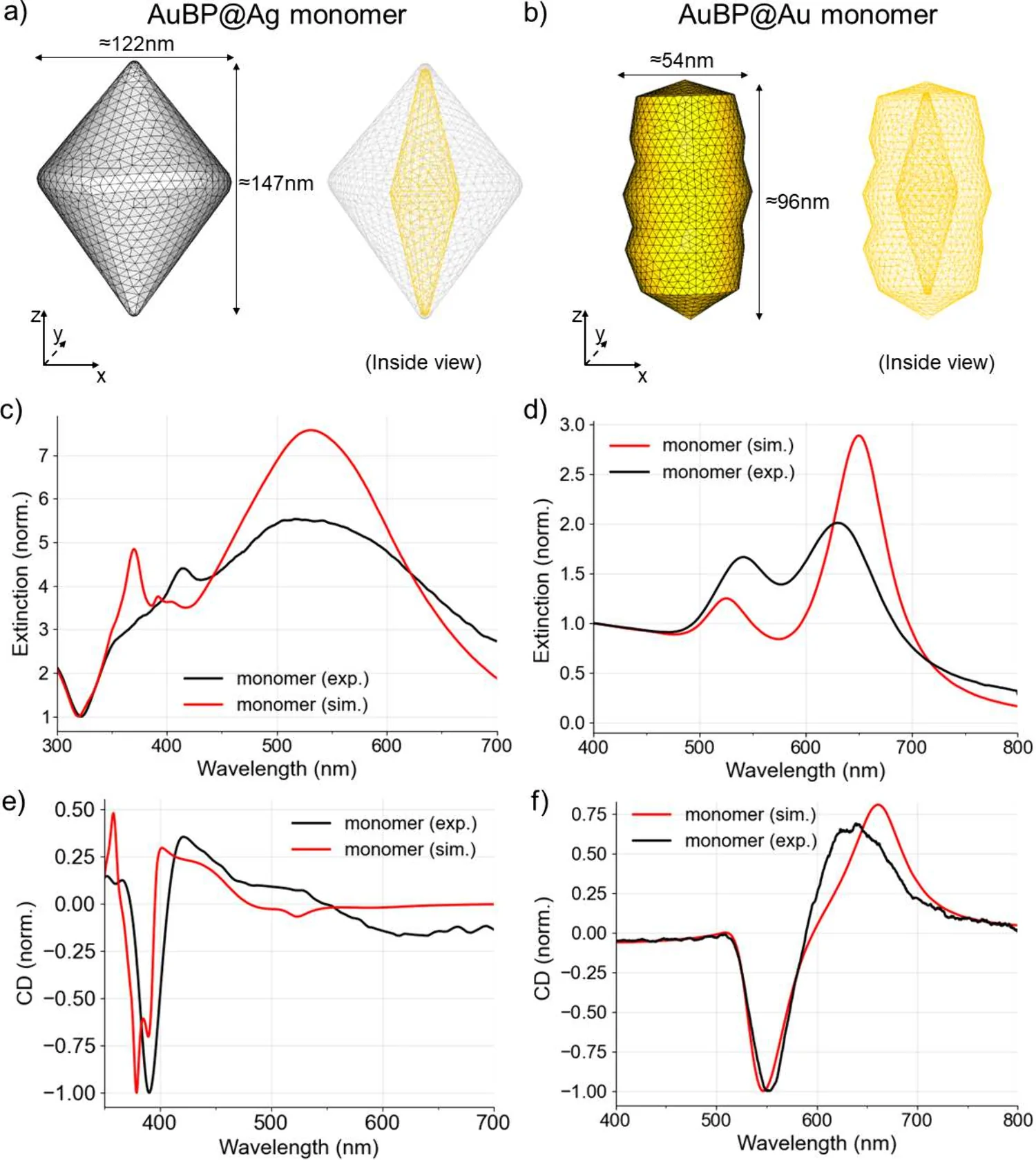
(a, b) Theoretical models of chiral AuBP@Ag and AuBP@Au NP monomers used for simulations. (c–f) The corresponding simulated (red) and experimental^29^ (black) normalized extinction spectra are illustrated in panels (c) and (d), while CD spectra are reported in panels (e) and (f), respectively.

In ref 29 aggregation was experimentally induced either by progressive surfactant dilution or by centrifugation at low surfactant concentration. TEM analysis of AuBP@Ag aggregated samples revealed that aggregates obtained under mild dilution conditions are mostly composed of dimers and trimers, while more complex longer chains of particles appear when aggregation is strongly promoted, e.g., by stronger dilution conditions or centrifugation. A similar aggregation trend is also observed in the present work for AuBP@Au aggregates (SI Section 2.9). We therefore decided to refer to these two regimes as mild and pronounced aggregation. The aggregation process was monitored in real time by UV–Vis spectroscopy to track the evolution of plasmonic coupling.^38^ Once the desired aggregation state is reached, the assemblies are encapsulated with a silica shell in colloidal suspension, preserving their structure by preventing further aggregation and rearrangement during TEM sample preparation (SI Section 2.7). According to ref 29, the degree of aggregation seemed to influence the intensity of the new CD peaks, but it did not have a significant effect on the peak position and shape. Moreover, electron microscopy analysis showed that small clusters, particularly dimers and trimers, are formed in mild aggregation condition (ref 29). Therefore, to balance computational cost with a realistic description of the aggregated samples, our computational analysis focuses on the simplest aggregates considered here, namely, dimers and trimers. Although this represents an approximation of the experimental systems, the main CD spectral features associated with aggregation remain qualitatively unchanged upon formation of larger assemblies. We therefore expect dimers and trimers to capture the essential optical effects induced by aggregation and to provide a reliable framework for interpreting the experimental observations. This simplified approach also enables a more efficient use of computational resources without compromising the main physical insights.

TEM images of the AuBP@Ag aggregates revealed^29^ that adjacent NPs in each aggregate show a preferential side-to-side contact with a relative twisting angle and an average face-to-face distance of 2 nm. The same arrangement is considered for the computational model of the AuBP@Ag dimers, as shown in Figure 2a. The case of AuBP@Au aggregates is discussed further below.

**Figure 2:**
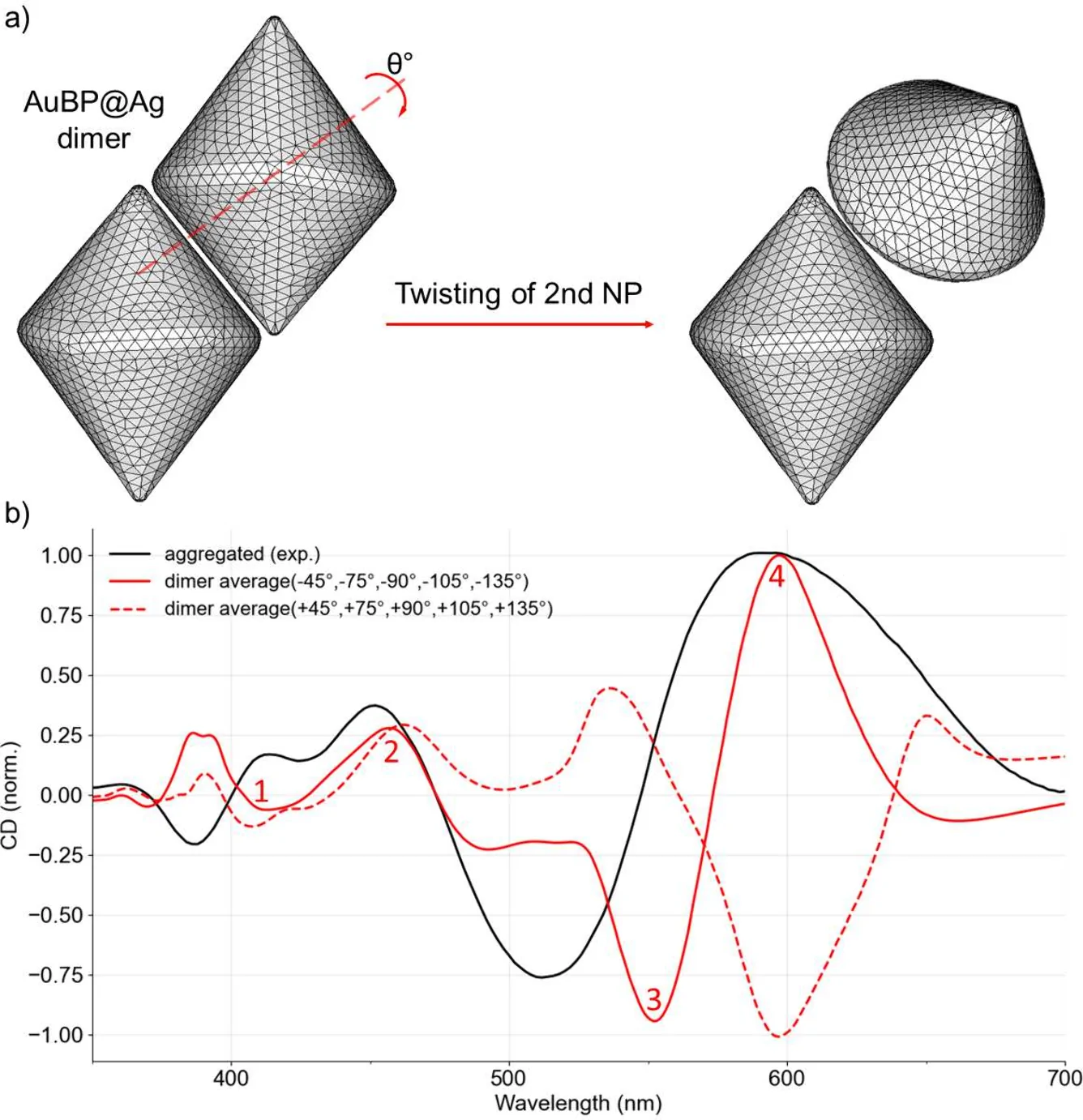
(a) Theoretical AuBP@Ag dimer model employed for simulations. (b) Simulated CD signal of the AuBP@Ag dimer model of panel (a) obtained by averaging the corresponding spectra of five different twisting angles: θ =  −45°, −75°, −90°, −105°, −135°(solid red). The overall CD spectrum is normalized by the intensity of the positive peak around 600 nm. The same normalization factor has been applied to the CD spectrum of the dimers featuring mirror twisting angles (red dashed line). The normalized experimental spectrum recorded for a sample with pronounced aggregation (solid black) is also shown.^29^ The surface charge distribution associated with each marked peak (red number) is shown in Figure 3

*AuBP@Ag Aggregates.* Moving from the single NPs to the aggregates, the main band of the experimental extinction spectrum centered at 550 nm decreases in intensity and it broadens. The same trend is observed in the simulations, moving from the monomer to the dimer model, as shown in Figure S3. The computed CD spectrum of the dimer is reported in Figure 2b (red lines), and it is compared with the experimental one of the aggregate (solid black). As no preferential twisting angle can be extracted from the TEM statistical analysis^29^ of the aggregated samples, the simulated spectra of the dimer have been averaged over different angle values. Notably, the intense CD features that appear around 550–600 nm upon aggregation can only be observed if there is some degree of twisting among NPs, regardless of the face-to-face dimer arrangement that is considered (different dimer arrangements and interparticle distances are also assessed, see Figures S4–S5). Indeed, if θ = 0°, those CD features completely vanish, as shown in Figure S6. It is even more remarkable that only a preferential twisting direction (solid red curve, Figure 2b) leads to dimers whose CD spectrum matches almost quantitatively with the experimental one. Indeed, the CD spectrum of the dimer featuring mirror twisting angles (dashed red) is almost the mirror spectrum, and their sum would make all CD features in the red-side of the spectrum vanish (the detailed CD contribution for each twisting angle is reported in Figure S7). Clearly, the experiments show that AuBP@Ag aggregation leads to a strong rise of CD signals in that part of the spectrum; therefore we conclude that chiral AuBP@Ag NPs must drive a chiral aggregation pattern, resulting in aggregates that feature a preferential twisting direction, which explains the observed new low-energy CD features. The same qualitative picture holds for a simple trimer model built by drawing inspiration from a trimer arrangement observed in TEM imaging, as shown in Figure S8. Inspection of the surface charge density associated with each plasmonic peak marked in Figure 2b reveals (see Figure 3) that the charge distribution on each monomer resembles, with slight modifications, the plasmonic modes of isolated AuBP@Ag monomers (see Figure S2).

**Figure 3:**
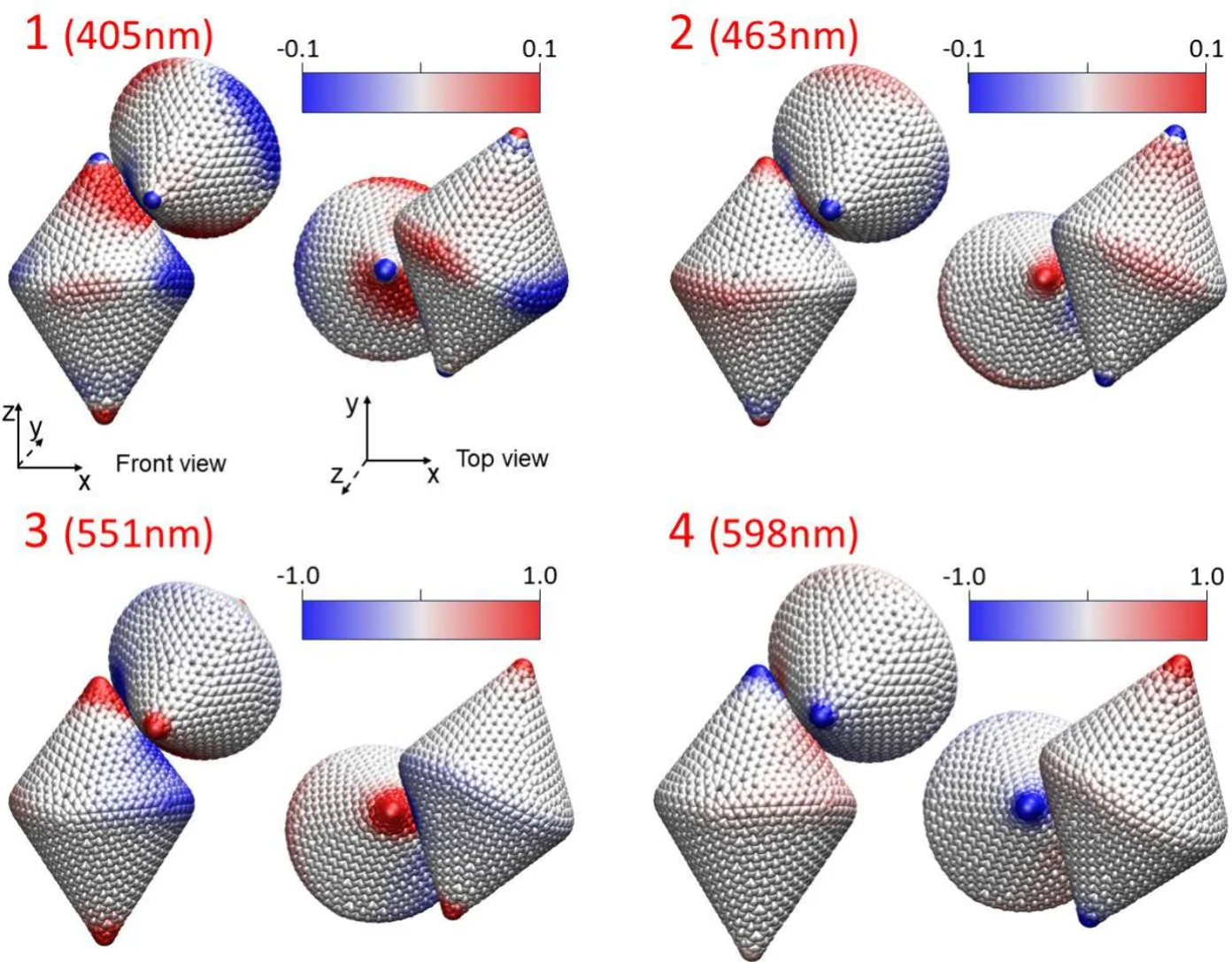
Dominant surface charge contribution induced by the EM field at the corresponding peaks marked in Figure 2 (see red numbers and corresponding wavelengths). Only the real part of the surface charge is shown. Each surface charge distribution is normalized to the modulus of the largest surface charge of the mode at 598 nm. The color scale of the weak high-energy modes (405,463 nm) is reduced to enhance visibility. Each white bead pictorially represents the centroid of the corresponding surface element of the discretized surface where the surface charge is located.

This indicates that these modes are largely preserved upon aggregation and become coupled within the dimer, giving rise to the observed spectral features. The high-energy region of the spectrum (below ≈500nm ) is primarily governed by multipolar modes of the individual monomers that interact upon dimer formation. Such multipolar contributions were previously shown to play a significant role also in the CD response of the monomers (ref ^28^). In contrast, the most intense low-energy peak, which emerges prominently upon aggregation in the experimental CD spectrum (around ≈600nm ), is characterized by charge distributions dominated by coupled dipolar oscillations between the two nanoparticles.

*AuBP@Au Aggregates.* Differently from AuBP@Ag, AuBP@Au NPs tend to aggregate distinctly, featuring a preferential tip-to-tip contact, as observed by TEM imaging^29^ (see also SI Section 2.9 and Figure S13). Based on this observation we built two different tip-to-tip models for the dimers, as illustrated in Figure 4a. Each model features a distance between the two NPs of the dimers of 2 nm, which is the average value resulting from TEM statistical data analysis performed over 300 particles (SI Section 2.9). In the T1 model the upper NP is tilted by a given angle θ with respect to the bottom NP, whereas in the T2 models the upper NP is tilted until the two lateral faces of the two NP tips facing each other are approximately parallel and 2 nm apart. The corresponding simulated CD spectra are reported in Figure 4b for different twisting angles.

**Figure 4:**
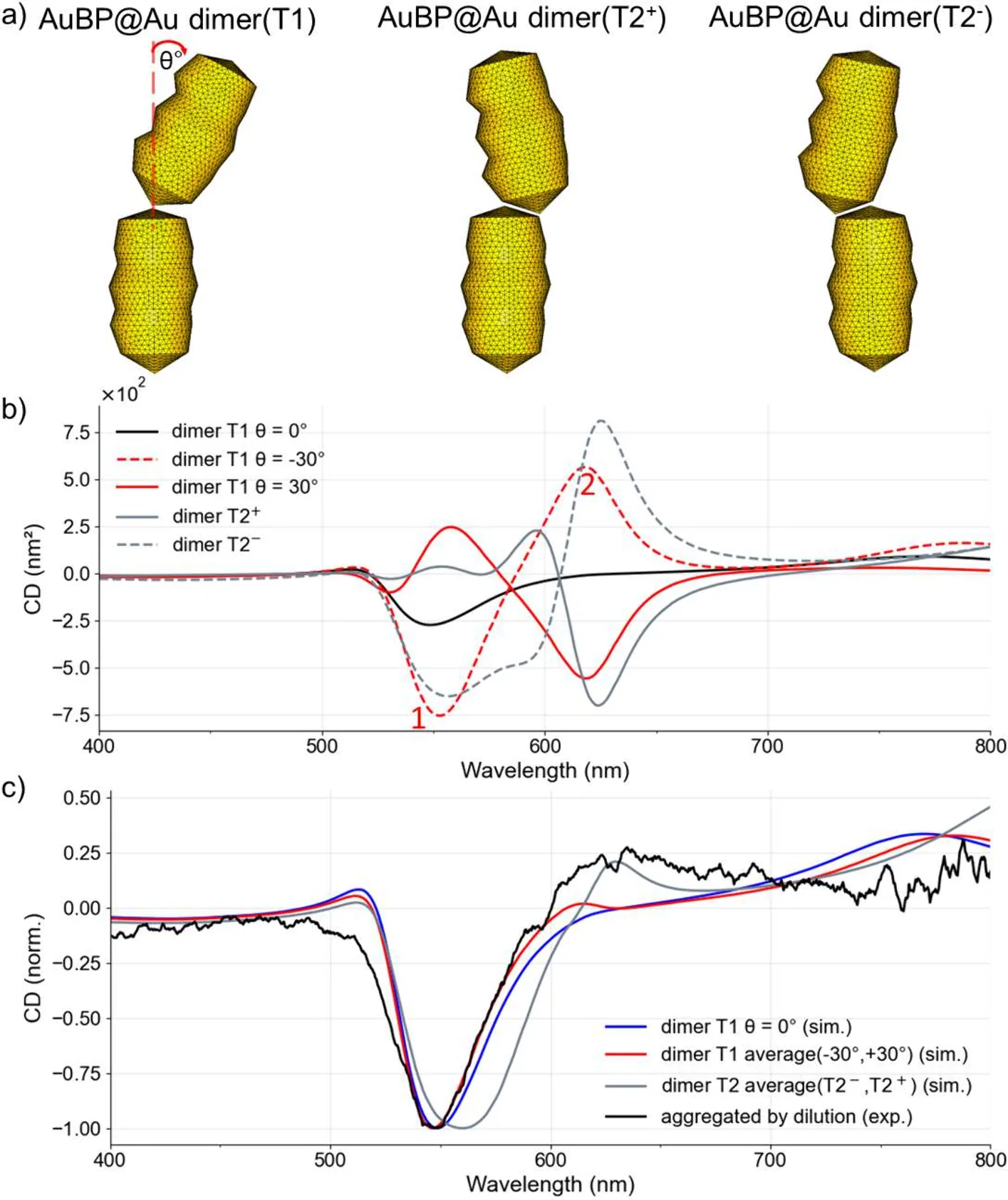
(a) Theoretical models of AuBP@Au NP dimers employed for simulations (b, c). (b) Simulated CD signal of the corresponding model dimers for different tilting angles. (c) Average of the corresponding CD spectra reported in (b) for dimer T1 with opposite tilt angles (solid red) and for dimers T2^+^ and T2^–^ (solid gray). The spectrum of model T1 with θ = 0° is the same as in panel (b). The experimental spectrum (solid black) is recorded for a sample with pronounced aggregation (SI Section 2). All spectra are normalized by the intensity of the corresponding negative peak located at ≈550 nm. The surface charge distribution associated with each marked peak (red number) is shown in Figure 5

In contrast to the AuBP@Ag case, the CD features of AuBP@Au dimers fall in the same spectral region of the monomers’, and no new low-energy CD peaks appear upon dimerization. By inspecting the surface change density (Figure 5) associated with each plasmonic peak marked in Figure 4b, it can be observed that similarly to AuBP@Au monomers,^28,29^ the CD peaks observed in the spectra of the dimers are related to dipolar transversal and longitudinal modes of the two NPs, and no multipolar modes contribution is observed.

**Figure 5:**
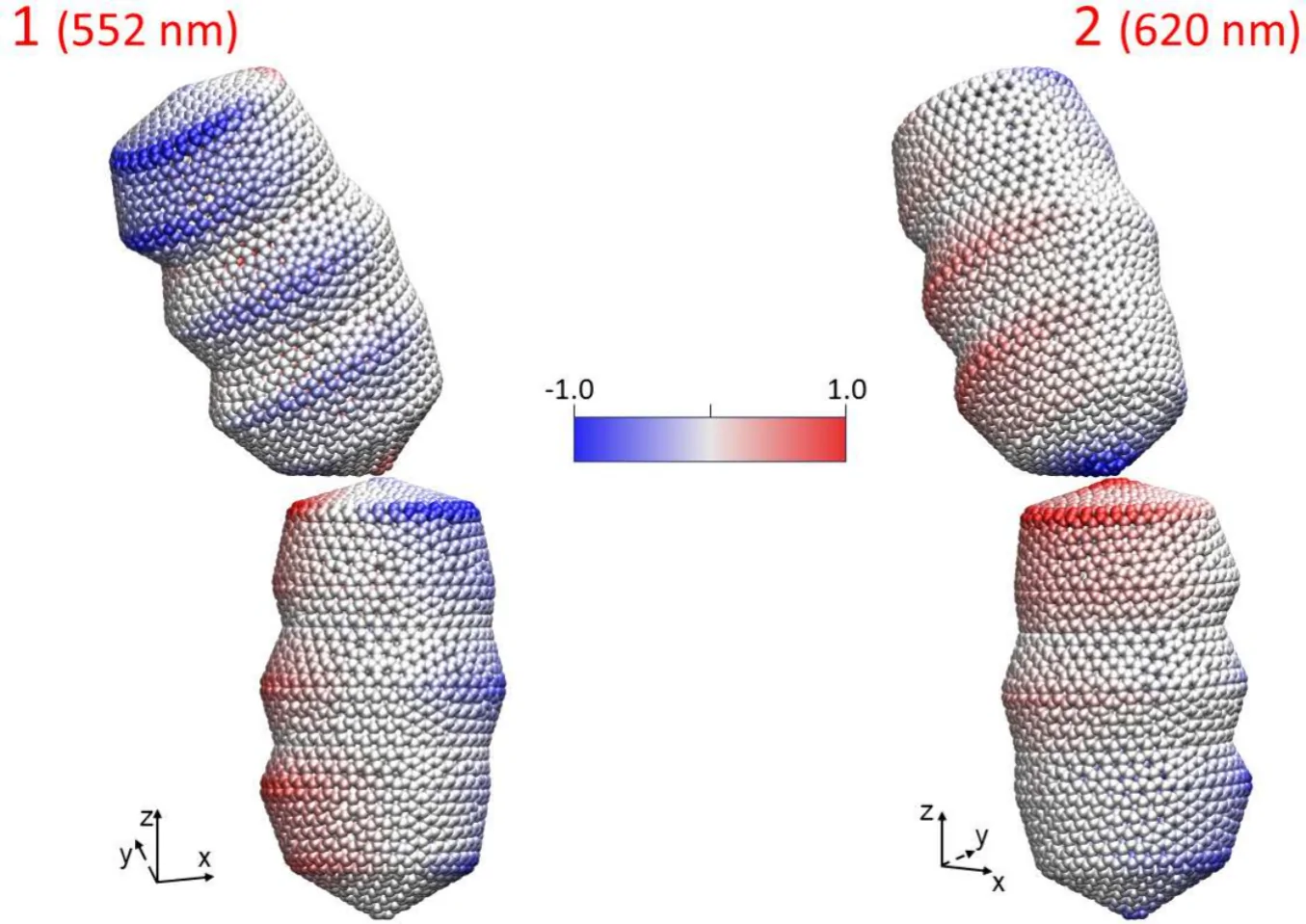
Dominant surface charge contribution induced by the EM field at the corresponding peaks marked in Figure 4b (see red numbers, related to the T1 model with θ = −30°). Only the real part of the surface charge is shown. Each surface charge distribution is normalized to the modulus of the largest surface charge of the mode at 620 nm.

Remarkably, either considering no twisting angle for model T1 (blue curve, Figure 4c) or by taking the average of the corresponding CD spectra with opposite twisting angle (red curve), the CD signal around 630 nm completely vanishes and only the higher energy peak located at ≈550 nm survives. This can be understood in light of the nature of the modes that contribute to the low-energy CD peak. Since the CD signal of AuBP@Au NPs is dominated by two dipolar charge oscillations along the longitudinal and transversal directions of the NPs, it is reasonable to expect that the longitudinal mode is mostly influenced by a tip-to-tip stacking configuration. Differently, side-to-side aggregation that would influence the transversal mode was not significantly observed experimentally under mild aggregation (SI Section 2.9). Similar stacking configurations and CD modulation effects have also been previously observed in aggregates of Au nanorods.^30,31,35^ Remarkably, the same trend, which is also confirmed by the T2 models (gray curve, Figure 4c), nicely match with the experiments, where mild aggregation is induced by dilution (black curve). Both the T1 and T2 models reproduce most of the key spectral features. However, the T1 model yields a lower root-mean-square deviation with respect to the normalized experimental spectrum (≈0.22) compared to the T2 model (≈0.28) , thereby indicating a slightly better agreement with the experimental data. Further experimental details are provided in Section SI 2. In light of these considerations we could also hypothesize about the origin of the complete CD suppression observed in previous experiments^29^ with pronounced aggregation and also reproduced here by studying the aggregation at different surfactant concentrations (Figures S14 and S15). Indeed, in that strong aggregation regime both tip-to-tip and side-to-side contacts may be promoted, thereby leading to CD depletion of the CD peak located at ≈550 nm as well, which is mostly dominated by transversal dipolar resonances (Figure 5). Based on these results we infer that AuBP@Au aggregation, unlike AuBP@Ag aggregation, does not lead to any preferential tilting handedness for the aggregates, as the experimental signal is correctly reproduced by simulations only if averaging over different tilting directions is performed. This emerging behavior in AuBP@Au aggregates turns out to be related to the different aggregation pattern of AuBP@Au particles (mainly tip-to-tip) with respect to AuBP@Ag NPs (mainly side-to-side). Indeed, if a similar aggregation pattern with relative twisting among adjacent particles is considered for AuBP@Au dimers as well, the resulting simulated CD signal (Figure S11) does feature low-energy peaks, similarly to the AuBP@Ag twisted dimers, which are however absent in the experimental data.

In this work, by combining continuum electrodynamics modeling and new ad-hoc experiments, we have shed light on the recently observed aggregation-induced modification of CD of chiral AuBP@Au and AuBP@Ag core–shell plasmonic NPs. A synergistic comparison between simulations and experimental data revealed that the different behavior of the CD response upon particle aggregation, either the appearance of new low-energy CD bands or CD suppression, is intimately related to the metal nature of the outer shell. Indeed, AuBP@Ag NPs, whose outer shell is made of silver, show the emergence of new low-energy CD signals upon aggregation that are absent at the monomer level. Such signals have been traced back to the formation of chiral aggregates of given handedness which arise as a result of side-to-side interactions among adjacent particles that feature a relative twisting angle. Such preferential arrangement eventually leads to chiral aggregates, thus explaining the observed CD response upon aggregation. On the contrary, AuBP@Au NPs made of a gold core and gold shell mostly show a tip-to-tip contact among particles in the aggregates. Based on this different stacking arrangement, the observed experimental CD response, which shows suppression of the low-energy CD peak of the monomers, is captured by the simulated models only if averaging over opposite tilting directions of Au NPs in the aggregates is performed, thereby suggesting that for AuBP@Au NPs no preferential handedness is observed upon aggregation. Despite the simplicity of our models, which consider only small aggregates and do not fully capture the heterogeneity of the experimental samples, our findings nevertheless reveal clear differences in the interactions governing chiral core–shell plasmonic nanoparticles. Importantly, the surfactant-induced aggregation process does not provide deterministic control over discrete aggregates with predefined orientations, but rather results in statistical distributions of monomers, dimers, trimers, and larger assemblies. Based on the experimentally observed aggregates, our results identify the underlying mechanisms governing the CD response and suggest that engineering aggregation pathways, particularly through nanoparticle surface morphology, could enable rational modulation of the plasmonic CD response. In particular, while the dominant contribution to the PCD response arises from the cluster geometry, this effect is closely intertwined with the morphology of the outer metal shell. AuBP@Ag nanoparticles, characterized by relatively extended lateral facets, naturally favor side-to-side aggregation, thereby suppressing tip-to-tip configurations. In contrast, AuBP@Au nanoparticles exhibit the characteristic *gnocchi*-like^28^ morphology and lack extended facets. As a result, their aggregates preferentially adopt tip-to-tip configurations, leading to the spectral changes discussed in the manuscript. While we cannot exclude the possibility that other factors, such as the different surfactant counterions (Br^–^ and Cl^–^), may influence the preferential tip-to-tip or side-to-side arrangement of the particles, our analysis suggests that the resulting CD response can be primarily rationalized in terms of the relative arrangement of the nanoparticles and that their morphology plays a central role in influencing the resulting PCD response of the aggregates. Although further investigations, potentially complemented by dedicated TEM tomography measurements, will be necessary to fully elucidate these effects, we believe that our findings open promising and largely unexplored perspectives for tuning PCD through the engineering of the exposed surface characteristics of individual chiral plasmonic building blocks.

## Supplementary Material



## Notes

Further computational and experimental details: monomer models; AuBP@Ag dimer model extinction spectrum; AuBP@Ag dimer stacking configurations and influence of gap distance; AuBP@Ag dimer model CD features and influence of twisting angle on CD; AuBP@Ag trimer model; AuBP@Au dimer models (extinction and CD spectra); synthesis and aggregation techniques of AuBP@Ag and AuBP@Au monomers and aggregates; characterization with TEM and optical measurements (PDF)
