# Supplementary material for: Deciphering Aggregation-Induced Tunable Circular Dichroism of AuBP@Ag and AuBP@Au Chiral Core–Shell Plasmonic Nanoparticles
Source: J Phys Chem Lett. 2026 Oct 15;17(43):12318–25. doi: 10.1021/acs.jpclett.6c02669 (PMC13726469; doi:10.1021/acs.jpclett.6c02669)
Supplement: Supplementary file 1 [file jz6c02669_si_001.pdf]

Supporting information: Deciphering Aggregation-Induced Tunable Circular Dichroism of AuBP@Ag and AuBP@Au Chiral Core-Shell Plasmonic Nanoparticles

Marco Romanelli,† Pablo Mariani,‡ Antonio Carone,‡,¶ Anthony Désert,‡ Stephane Parola,‡ Stefano Corni,†,§,∥ and Ivan Rivalta*,⊥,#

†*Department of Chemical Sciences, University of Padova, via Marzolo 1, 35131 Padova, Italy*

‡*École Normale Supérieure de Lyon, CNRS, Université Claude Bernard Lyon 1, Laboratoire de Chimie, UMR 5182, 46 Allée d'Italie, 69364 Lyon, France*

¶*Sustainable and Bio-inspired Materials, Max Planck Institute of Colloids and Interfaces, Science Park Golm, 14476 Potsdam, Germany*

§*CNR Institute of Nanoscience, via Campi 213/A, 41125 Modena, Italy*

∥*Padua Quantum Technologies Research Center, University of Padova, 35131 Padova, Italy*

⊥*Department of Industrial Chemistry "Toso Montanari", University of Bologna, Viale del Risorgimento 4, 40136, Bologna, Italy*

#*Center for Chemical Catalysis – C3, University of Bologna, via Piero Gobetti 85, 40129 Bologna, Italy*

E-mail: i.rivalta@unibo.it

Contents

1 Computational details **3**

1.1 AuBP@Ag and AuBP@Au monomer models 4

1.2 AuBP@Ag dimer models 8

1.3 AuBP@Ag trimer model 13

1.4 AuBP@Au dimer models 14

2 Experimental details **17**

2.1 Chemicals 17

2.2 Synthesis of gold nanobipyramids AuBPs 17

2.3 Synthesis of chiral gold AuBP@Au NPs (adapted from ref.[1]) 17

2.4 Synthesis of chiral silver AuBP@Ag NPs (adapted from ref.[1]) 18

2.5 Self-assembly of chiral AuBP@Au NPs (adapted from ref.[2]) 18

2.6 Self-assembly of chiral AuBP@Ag NPs (adapted from ref.[2]) 18

2.7 Silicate encapsulation for TEM imaging (adapted from refs.[2,3]) 18

2.8 Characterization 19

2.9 Additional supporting experimental data for AuBP@Au aggregates 20

References **24**

1 Computational details

All computations presented in this work have been performed by using the MNPBEM[4] Matlab Toolbox which features a Boundary Element Method (BEM) numerical solution of classical macroscopic Maxwell equations for metallic nanoparticles embedded in homogeneous media and subjected to external perturbations (e.g. electromagnetic driving fields). In this work full-Maxwell (or retarded) simulations are performed.
The extinction spectra that are reported in both main text and SI have been obtained by computing the extinction cross-section of the corresponding model (monomer, dimer or trimer) by averaging the three extinction cross-sections that arise from exciting the system with an electric field which is respectively oriented along one of the three directions z,x,y and so the corresponding wave travels along x,y,z (see Fig. 1, main text, for the reference frame). In other words, the isotropic extinction is simulated and compared with the experimental one, whose sample contain different particles randomly oriented.
As for CD signals, the reported CD spectra consist of the average of three cross section values, each one related to a given wavector direction and representing the difference between left-handed and right-handed circularly polarized light for the same wavector direction. Cross-sections are usually given in nm^2 unless they are normalized to a given value. In the latter case specification is given.

The dielectric function of Au and Ag are taken from P. B. Johnson and R. W. Christy[5] and the refractive index of the surrounding medium has been set to 1.33 to account for the water environment considered in the experiments. The model does not account for possible modifications of the local dielectric environment induced by aggregation. If present, such effects may contribute to the observed discrepancies between simulated and experimental spectra, particularly in terms of peak positions and line broadening. However, these dielectric variations are expected to influence left- and right-circularly polarized light absorption almost equally and therefore should not significantly influence the CD response, which primarily originates from chiral electromagnetic coupling within the nanoparticle assemblies.

1.1 AuBP@Ag and AuBP@Au monomer models

The computational monomer models illustrated in Fig.S1 have been created by following the same procedure introduced in our previous works.[1,6] In essence, the AuBP@Ag monomer model is based on two nested surface meshes (see also Fig. 1a, main text) formed by two bi-cones with spherical tips,[6] representing the inner Au core and the outer Ag shell, respectively. The inner Au core features a length of 146 nm and width of 32 nm whereas the outer Ag mesh features a length of 147 nm and a width of 122.5 nm (aspect ratio 1.2). The model thus built is achiral, therefore, in order to make it chiral a slight deformation is applied (Fig. S1a). More precisely, the round base is asymmetrically deformed by alternatively translating upward (+z) and downward (-z) 5 consecutive points that correspond to the vertices of the pentagon that can be inscribed in the circle base. The magnitude of each translation is chosen by picking random different values in the range 1-3 nm to make sure that all possible symmetry planes are removed upon deformation. The same asymmetrical deformation is applied to the tips of the bipyramid, where the upper tip is translated by 1 nm along +z whereas the lower tip is translated by 0.5 nm along +z. Upon such deformation the monomer becomes chiral and the corresponding simulated CD spectrum nicely match with the experimental one (Fig. 1, main text). We note that in this work we deliberately avoid the spiral numerical "trick" introduced in Ref.[1] to enforce chirality in the outer surface mesh. In that approach, chirality is imposed artificially through a spiral tessellation pattern and therefore does not encode any physically meaningful structural information related to geometrical chirality. Although the method reproduces the energies of the CD peaks reasonably well, it also substantially increases the computational cost, since achieving chirality through spiral tessellation requires a much finer surface discretization with a significantly larger number of surface tesserae. Given that the chirally deformed structure shown in Fig.,S1a already reproduces the same main CD spectral features well without resorting to the spiral numerical "trick," in the present work we restrict our analysis to this model only.

The model for the AuBP@Au monomer (Fig. S1b) is the same that was introduced in our

previous work[1] to model the AuBP@Au gnocchi-like particles with jagged lateral surface. The outer surface mesh is 96 nm long and 54 nm wide (aspect ratio of 1.8), whereas the inner Au mesh is 84 nm long and 26 nm wide. Similarly to the AuBP@Ag case, even the AuBP@Au model thus created is achiral, so to make it chiral we have slightly deformed it asymmetrically by stretching the lateral "teeth" oriented along x and y by small random different values in the range ±1-3 nm along the corresponding directions. The same asymmetrical deformation has been applied to the tips by translating the upper tip by 1 nm along +z and the bottom tip by 0.5 nm along +z to remove all possible symmetry elements. The resulting structure is chiral and the simulated CD spectrum (Fig. 1, main text) well match with the experimental CD features.

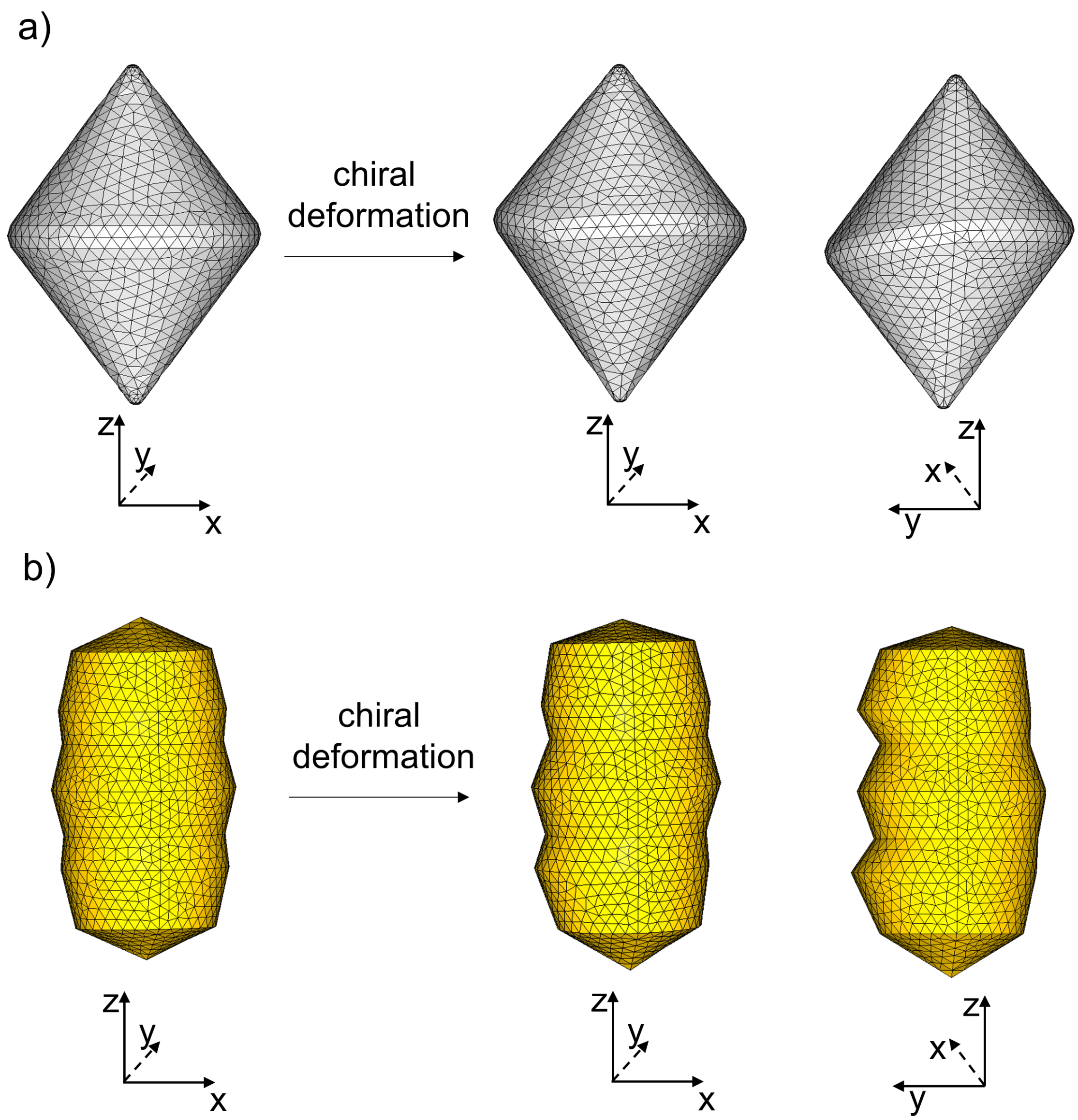

Figure S1: a) AuBP@Ag and b) AuBP@Au single NP computational models before and after geometrical distortion which is needed to create a chiral model.

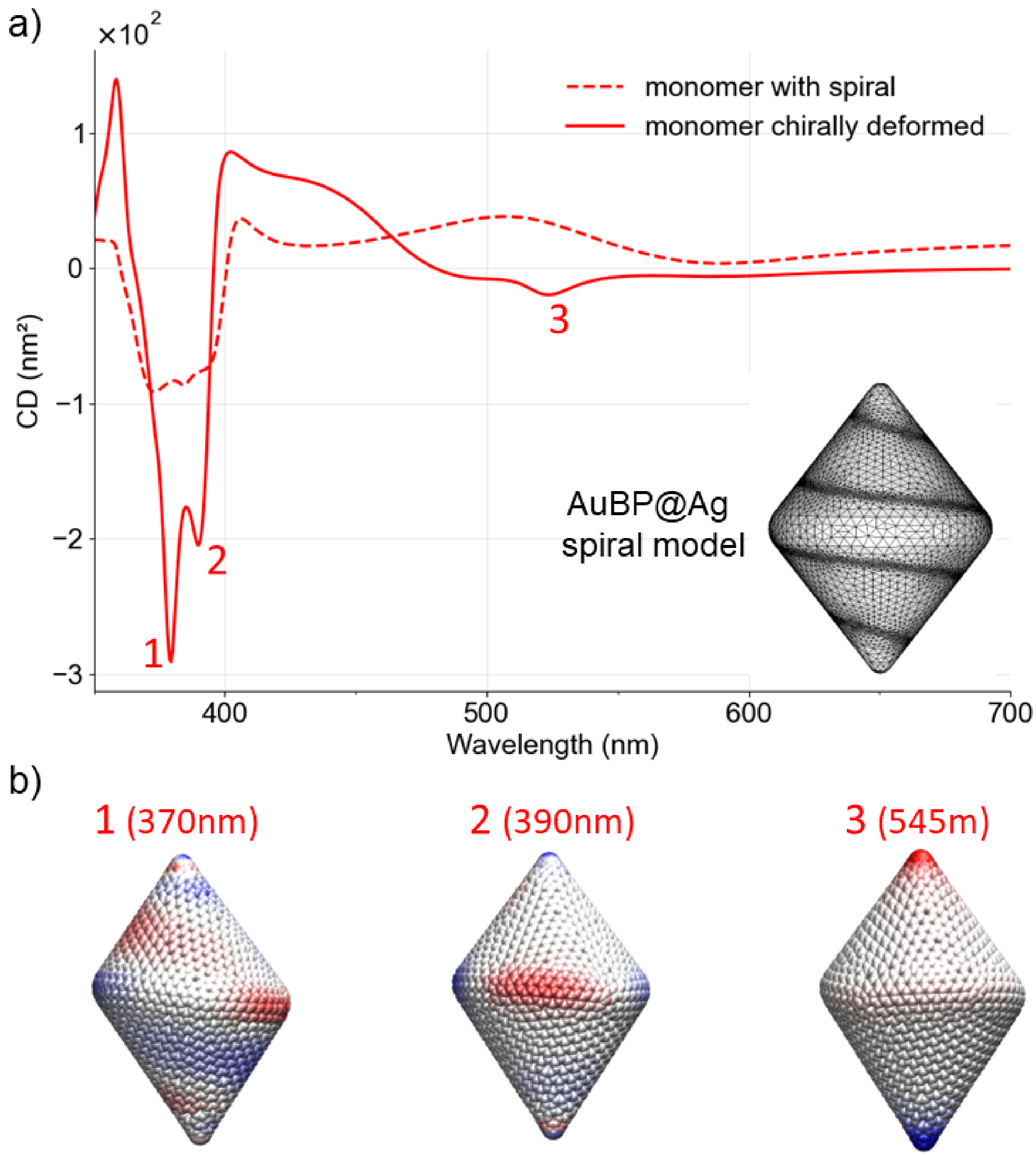

Figure S2: a) Comparison between the simulated CD spectra of the AuBP@Ag chirally deformed model of Fig. S1a (solid red) and the AuBP@Ag monomer featuring a spirally thickened surface mesh (dashed red), as depicted in the inset (see also ref.[1]). The surface charge distribution of the chirally deformed model associated to each marked peak (red number) is shown in panel b.

1.2 AuBP@Ag dimer models

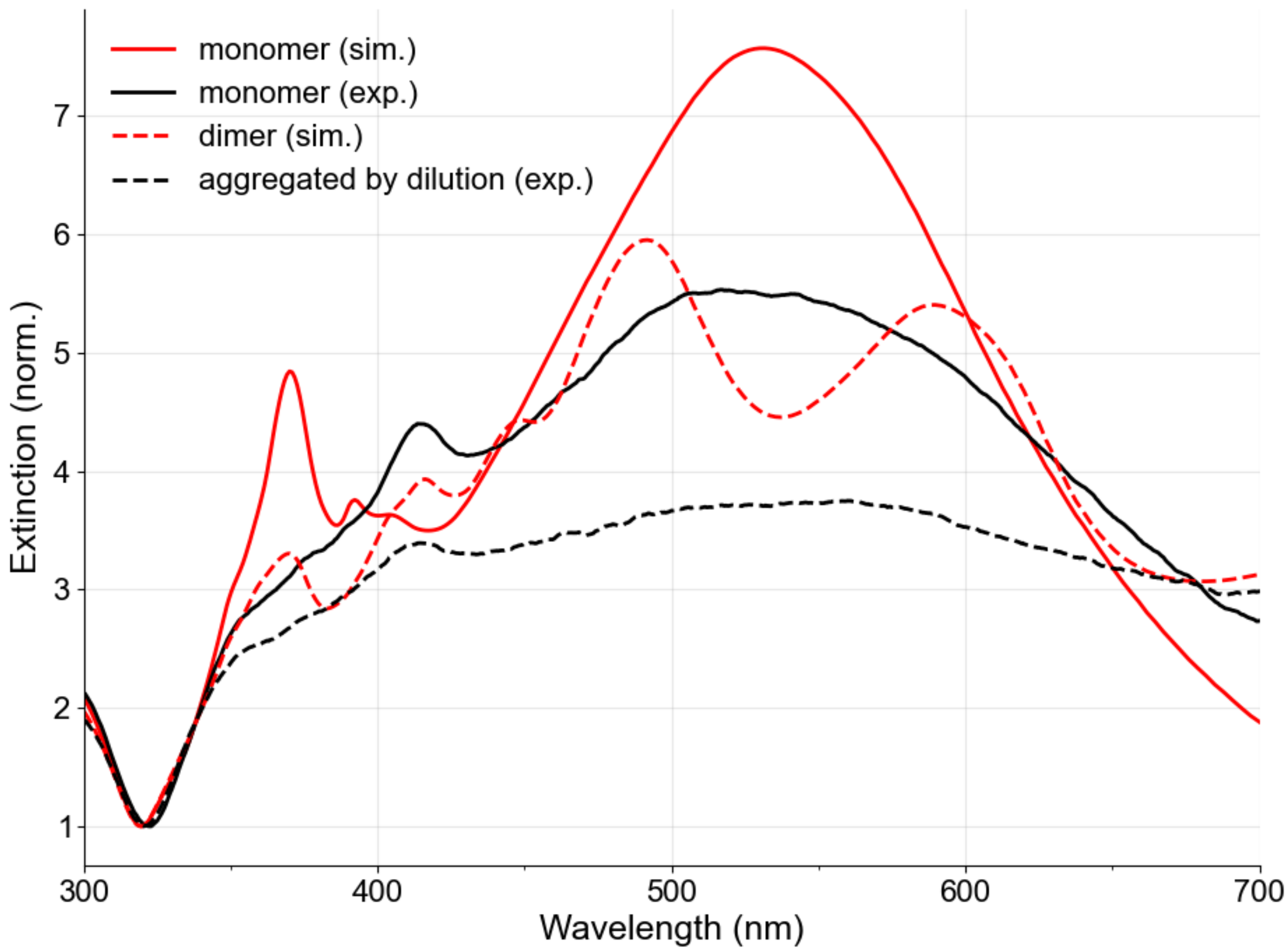

Figure S3: Simulated extinction spectrum of the AuBP@Ag monomer model illustrated in Fig. 1a (solid red) and AuBP@Ag dimer model shown in Fig. 2a (dashed red). The reported spectrum of the dimer is obtained by averaging the corresponding spectra of three different twisting angles: $\theta = -90°$, -75°, -105°. Experimental extinction spectra of AuBP@Ag nanoparticles before (solid black) and after (dashed black) aggregation by dilution are also shown. The original sample has been diluted to the CTAC concentration of 0.005mM, see ref.[2]

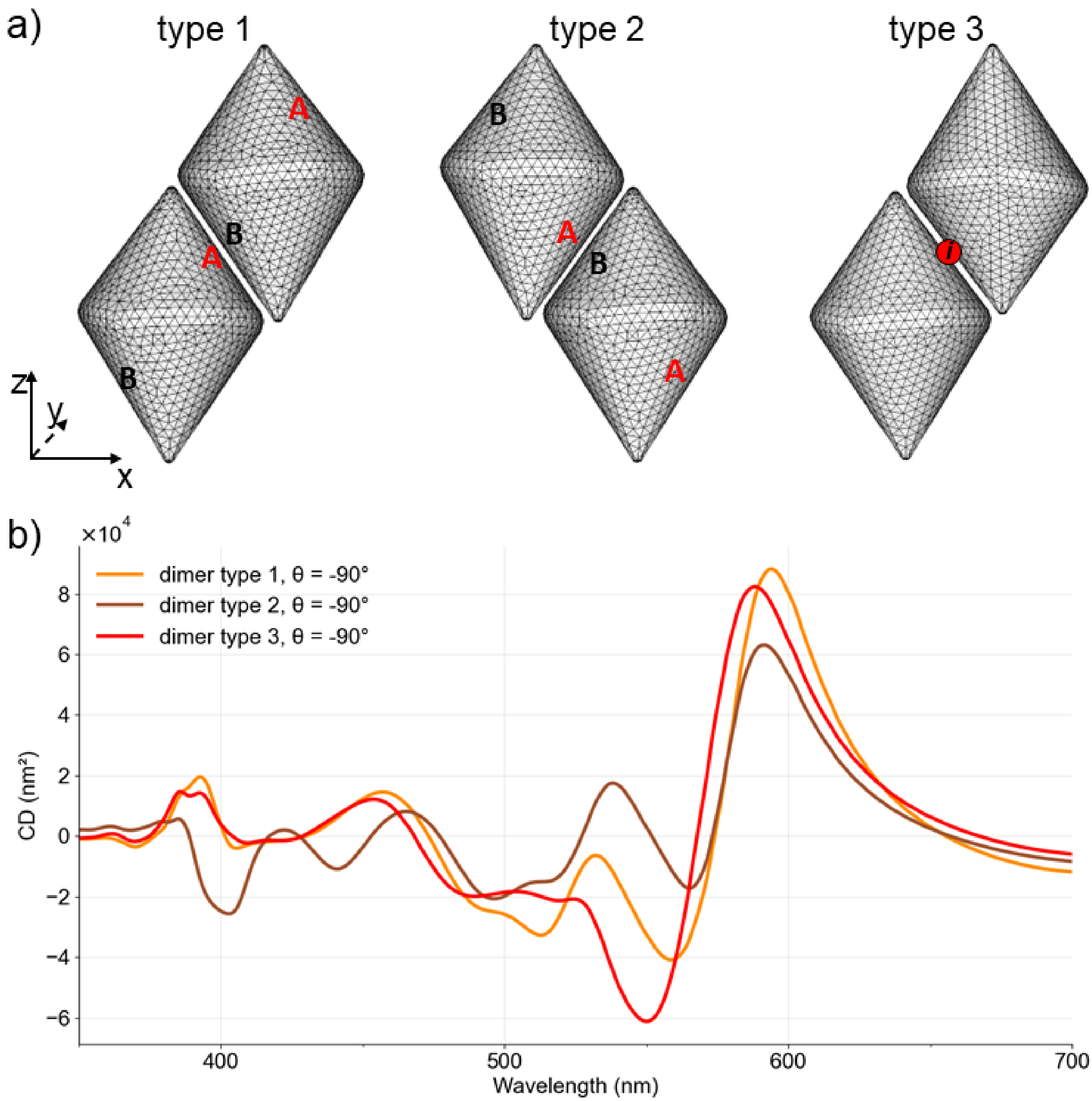

Figure S4: a) Three different AuBP@Ag dimer arrangements that have been tested. The type 1 dimer has been created by translating a copy of the AuBP@Ag bottom monomer to the top right corner, while the type 2 model has been obtained by translation to the top-left side. The colored letters indicate the corresponding surfaces upon translation. The type 3 dimer instead, which is also the same of Fig.2a main text, has been created such that the two NPs within the dimer can be exchanged approximately by inversion symmetry around the marked point. b) Simulated CD spectra of the dimers reported in a) upon twisting the second BP of -90° with respect to the face-to-face normal direction, as depicted in Fig. 2a main text.

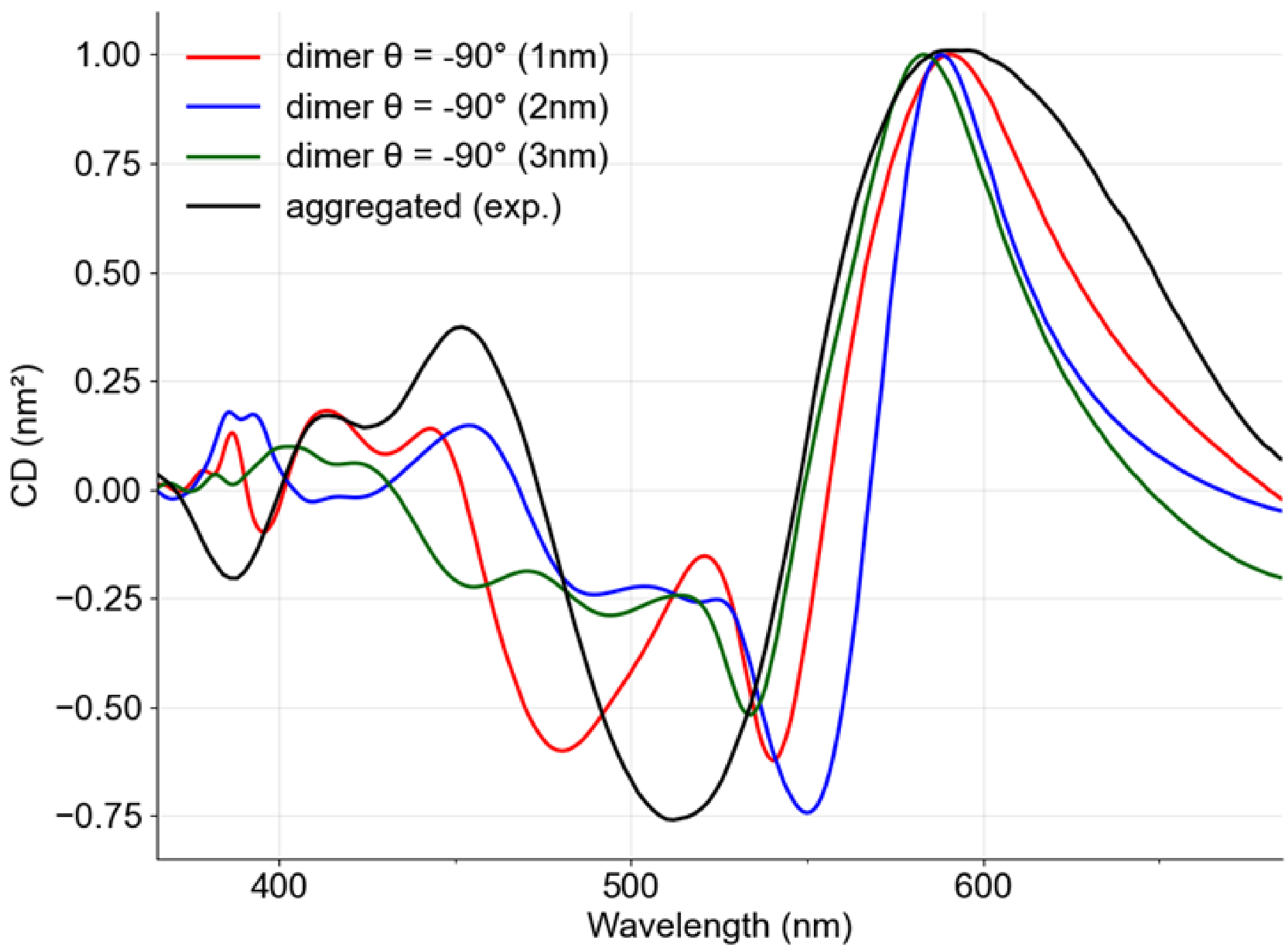

Figure S5: Simulated CD spectra of the AuBP@Ag dimer model of Fig.2a (type 3) with a twisting angle of -90° and interparticle distance of 1 nm (red), 2 nm (blue) and 3 nm(green). The experimental CD spectrum acquired after sample aggregation by centrifugation is also shown (solid black).

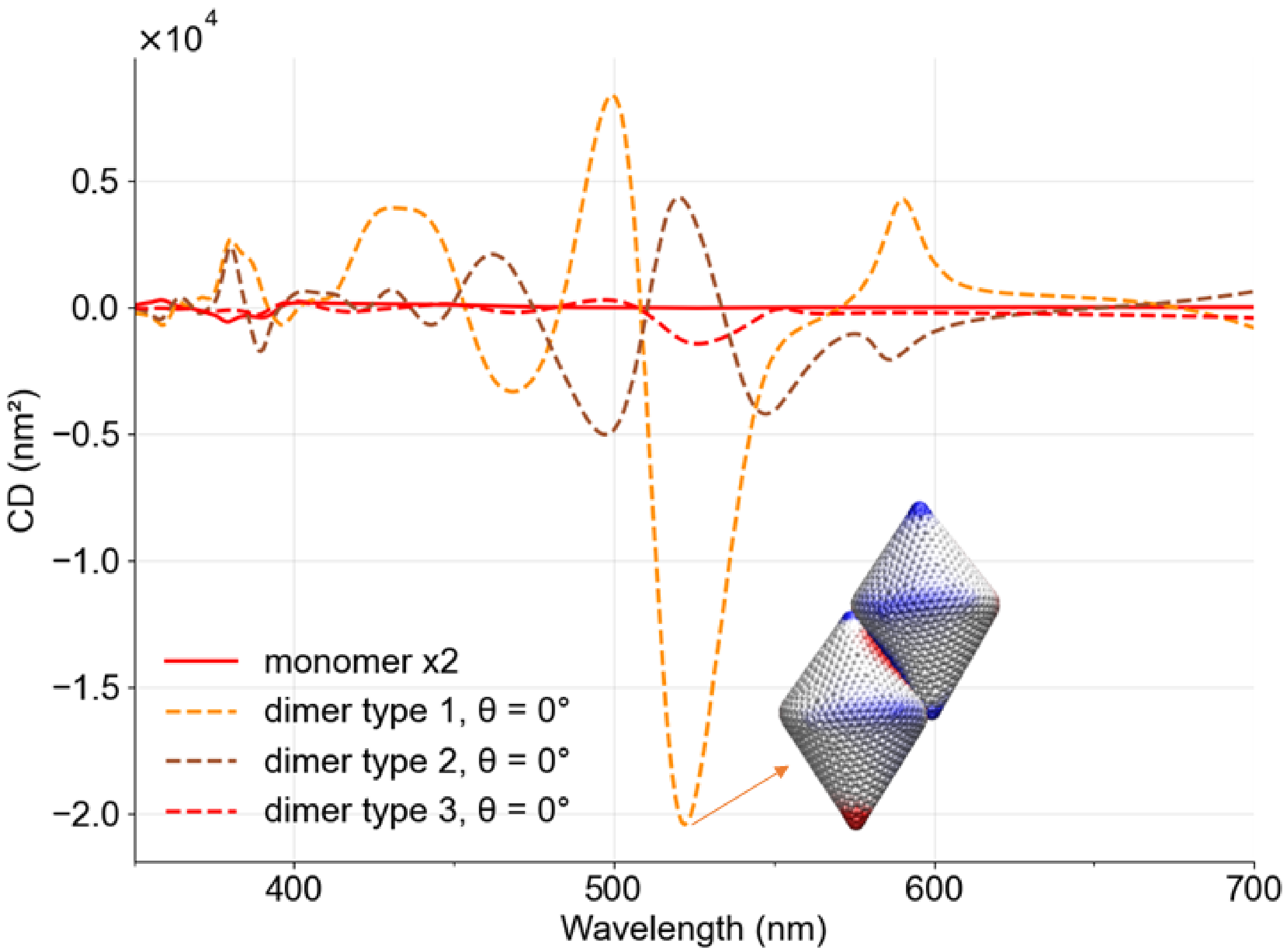

Figure S6: Simulated CD spectra of the AuBP@Ag dimer type 1 (dashed orange), type 2 (dashed brown) and type 3 (dashed red) of Fig. S3 with no twisting angle, $\theta = 0°$. The solid red spectrum is the computed CD signal of the monomer model scaled by a factor of 2.

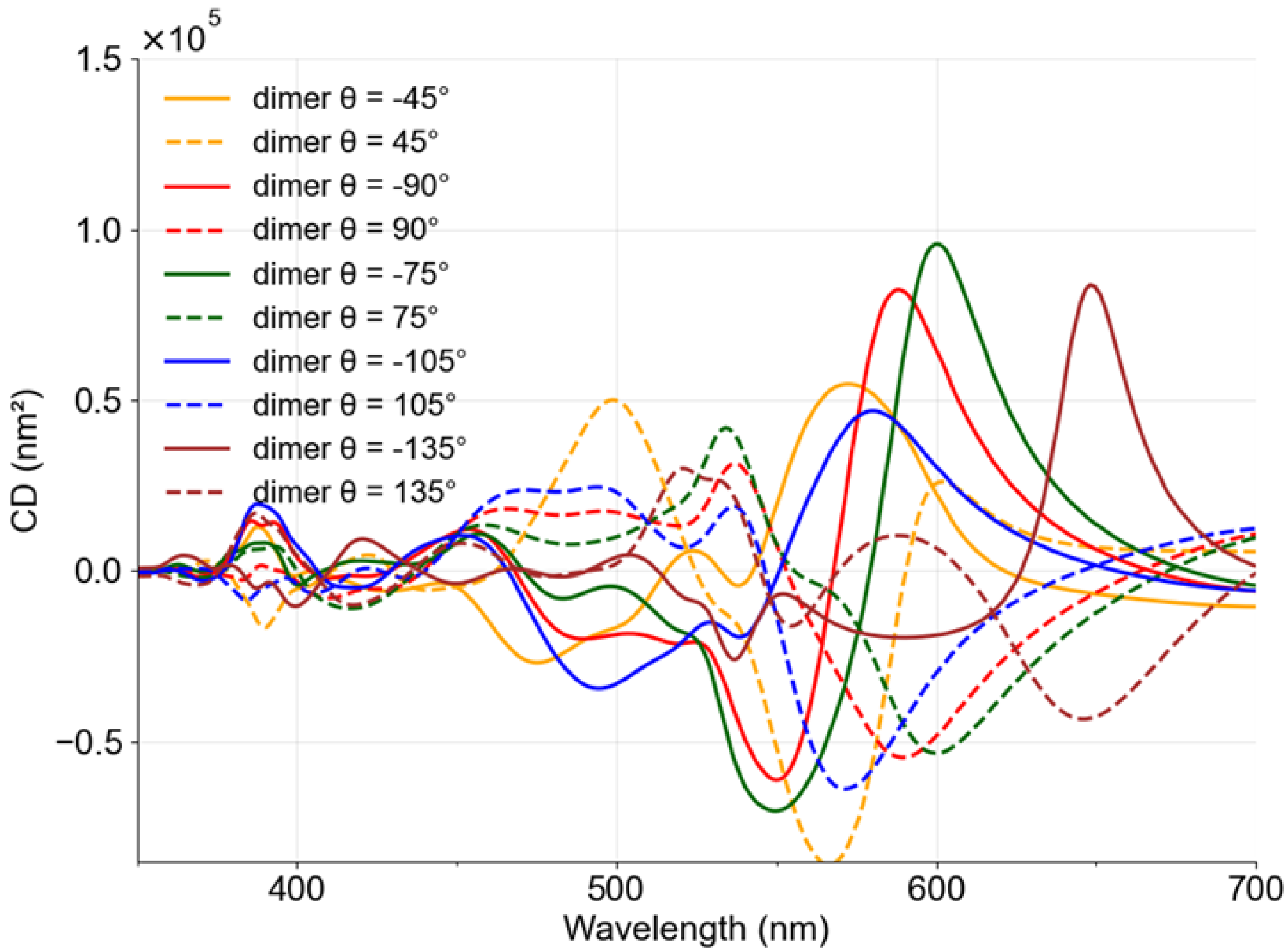

Figure S7: Simulated CD spectra of the AuBP@Ag dimer model of Fig.2a (type 3) for different twisting angles.

1.3 AuBP@Ag trimer model

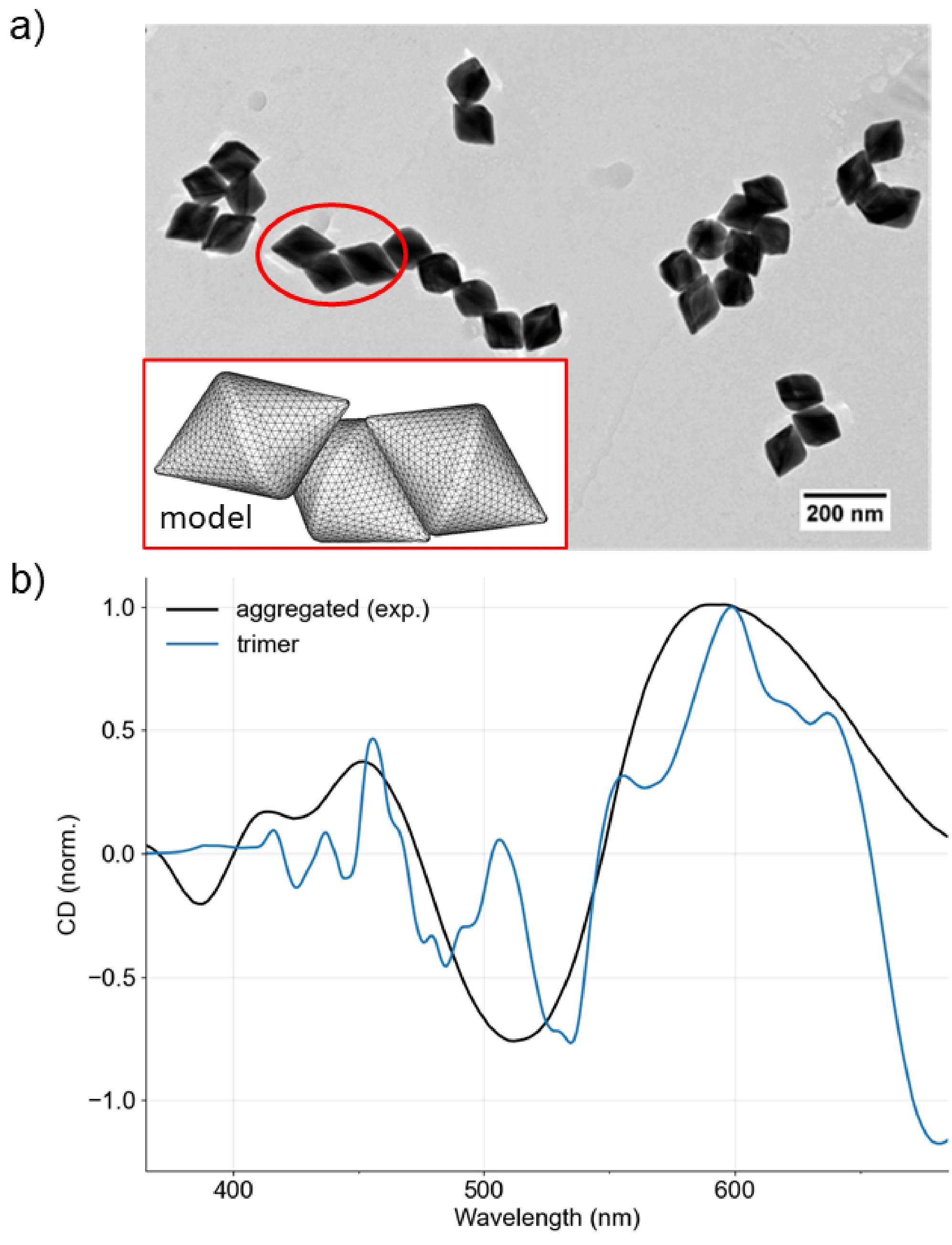

Figure S8: a) TEM image of AuBP@Ag particle aggregates after sample centrifugation, adapted from.[2] The red inset shows a trimer model built taking inspiration from the corresponding TEM image, see red circle. The trimer model is constructed by using the type 3 arrangement (see Fig. S4). b) Simulated CD spectrum of the trimer toy model (solid blue) normalized by its maximum intensity ($\approx$ 600 nm). A constant shift of 25 nm towards lower energies has been applied to the simulated CD raw data. The experimental CD spectrum acquired after sample aggregation by centrifugation is also shown (solid black).

1.4 AuBP@Au dimer models

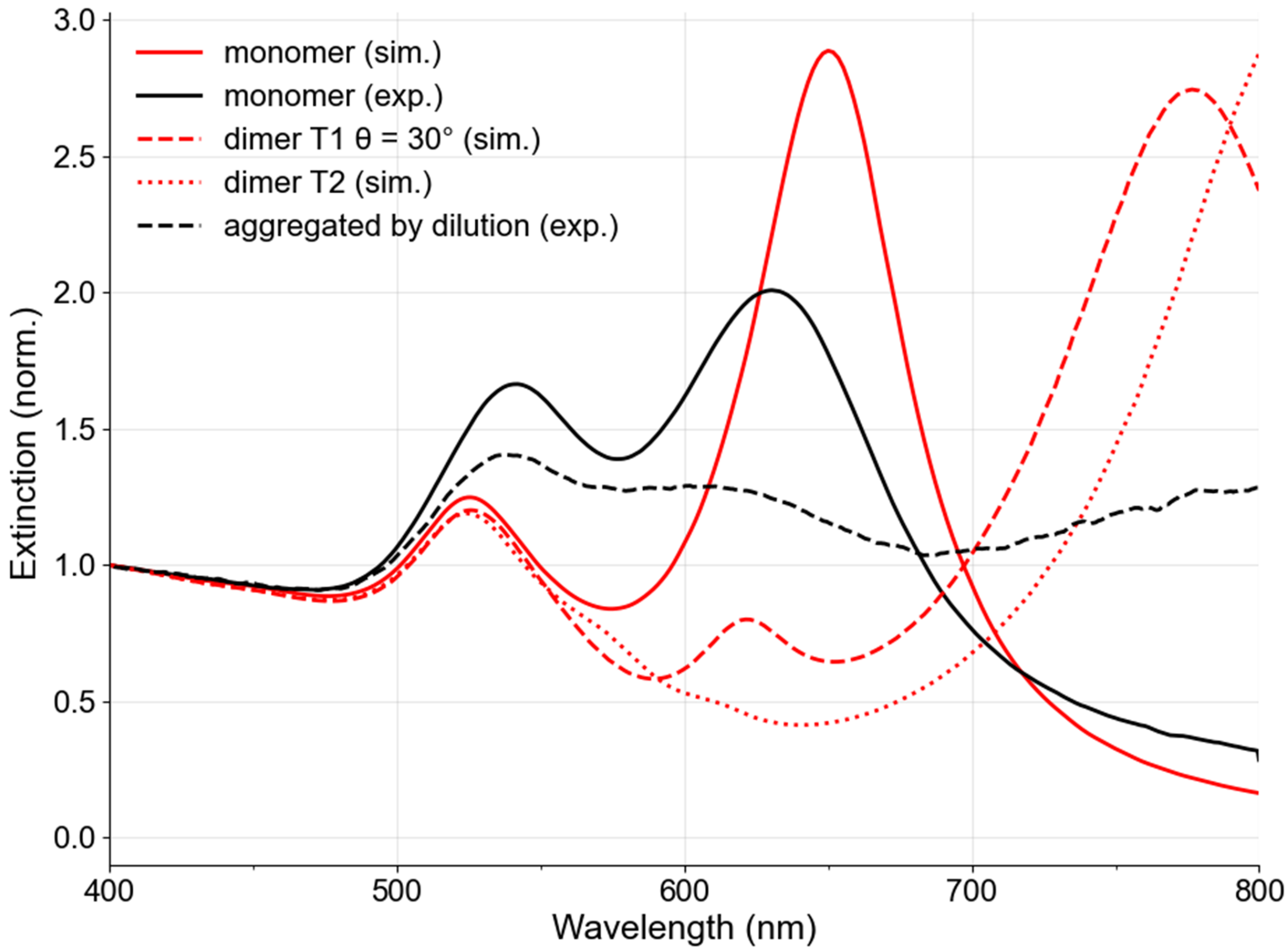

Figure S9: Simulated extinction spectrum of the AuBP@Au monomer model of Fig.1b (solid red) and of the AuBP@Au T1 and T2 dimer models of Fig.3a (dashed and dotted red, respectively). Experimental extinction spectra of AuBP@Au nanoparticles before (solid black) and after (dashed black) aggregation by dilution are also shown. The original sample has been diluted to the CTAC concentration of 0.005mM, see ref.[2] and SI 2.

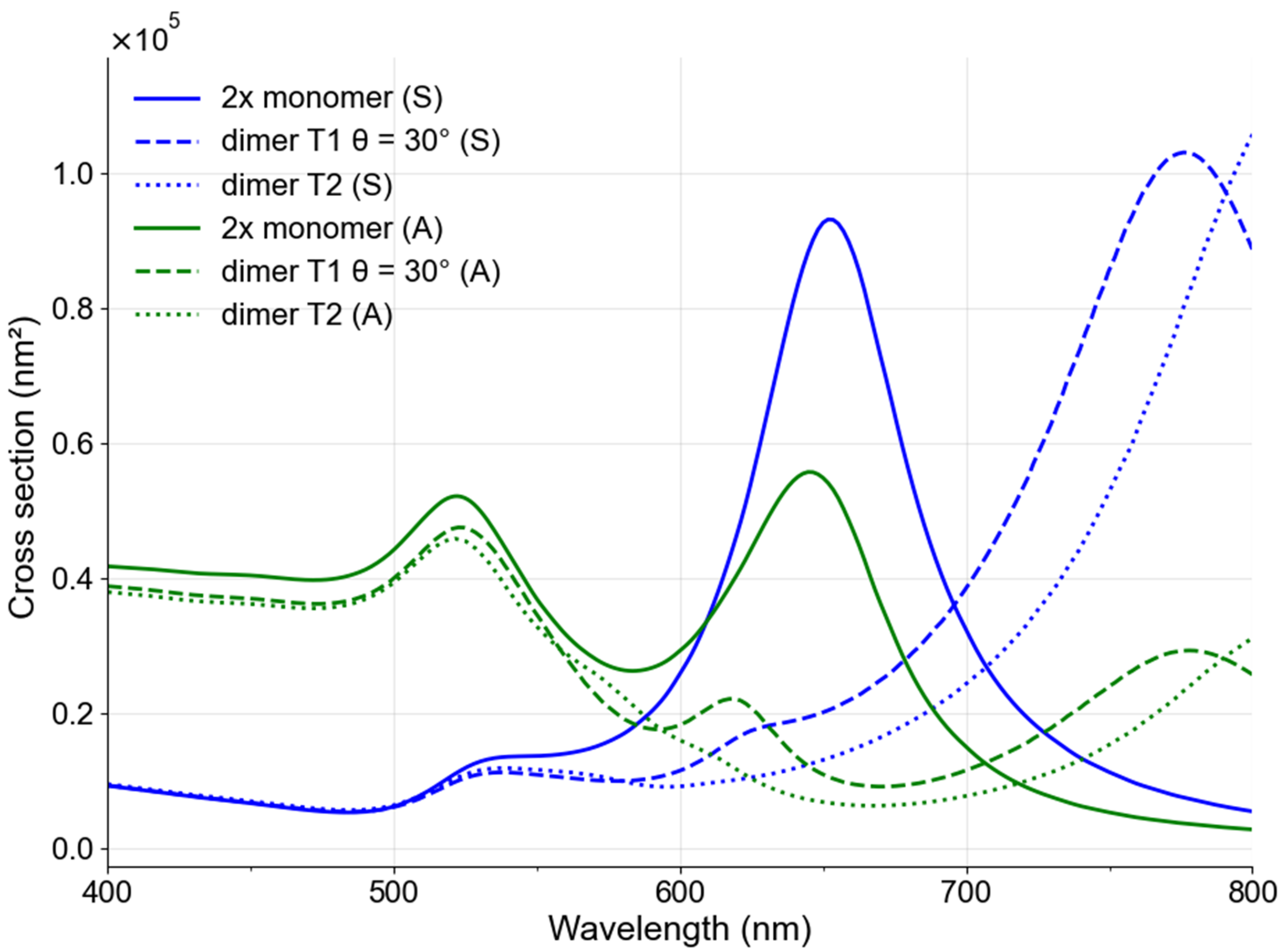

Figure S10: Corresponding scattering (blue) and absorption (green) contributions to the simulated extinction cross sections reported in Fig. S9. Solid lines refer to monomer data, dashed lines refer to model dimer T1 and dotted lines refer to dimer model T2.

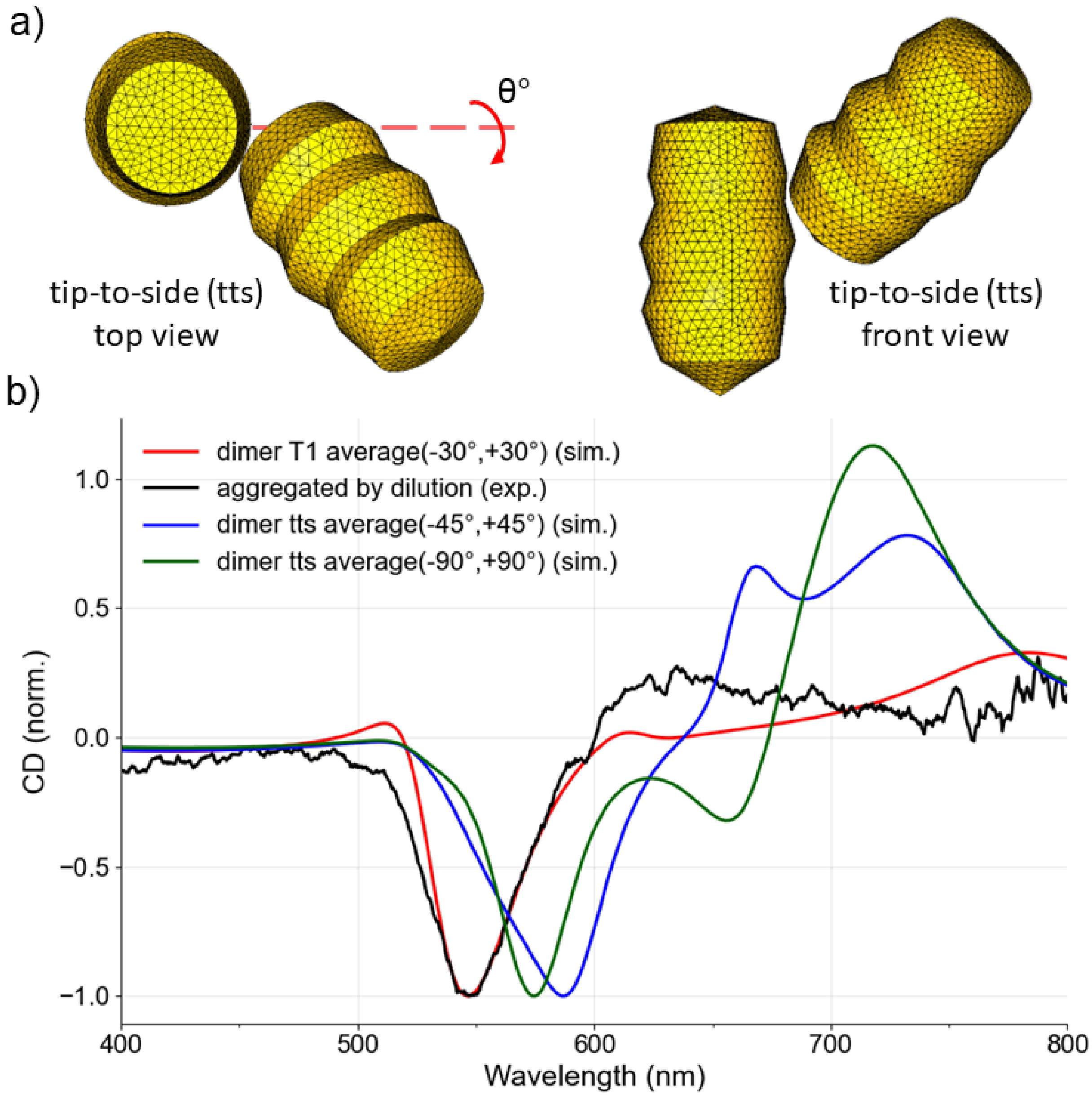

Figure S11: a) tip-to-side AuBP@Au dimers featuring relative twisting angle θ between the two NPs. b) Average of the simulated CD spectra of tts dimers (panel a) with $\theta = \pm\ 45^\circ$ (blue) and $\theta = \pm\ 90^\circ$ (green). The red spectrum is the same as in Fig. 3c main text (tip-to-tip model T1), while the black spectrum is the experimental one, as in Fig. 3c.

2 Experimental details

2.1 Chemicals

Anhydrous potassium silicate (SiO_2:K_2O 2.5:1 wt%), silver nitrate ($AgNO_3$, 99.9995%) and tetrachloroauric acid trihydrate ($HAuCl_4 \cdot 3H_2O$, 99.99%) were purchased from Alfa Aesar. Hexadecyltrimethylammonium chloride solution (CTAC, 25 wt% in water), L-ascorbic acid (99%), sodium borohydride ($NaBH_4$, 98%) and 8-hydroxyquinoline (99%, recrystallized) were purchased from Sigma-Aldrich. Hexadecyltrimethylammonium bromide (CTAB, 99%) and L-cysteine (L-Cys, 98%) were purchased from Acros Organics. D-cysteine (D-Cys, 98%) was purchased from ChemCruz. Sodium hydroxide (NaOH, 97%) and absolute ethanol were purchased from Carlo Erba. All aqueous solutions were prepared in Milli-Q distilled water (16-17 $M\Omega \cdot cm$).

2.2 Synthesis of gold nanobipyramids AuBPs

The synthesis of AuBPs is fully described in ref.[7] Two batches were synthesized, with lengths of 84 nm and 147 nm, used as seeds in the following syntheses of chiral AuBP@Au and AuBP@Ag nanoparticles, respectively.

2.3 Synthesis of chiral gold AuBP@Au NPs (adapted from ref.[1])

In a vial, 1 mL of 100 mM CTAB solution was mixed with 50 µL of AuBPs diluted suspension ($[Au^0]_m^{400nm}$ = 0.5 g/L) and 2 µL of fresh L or D-Cys (10 mM). After 30-40 minutes of incubation at room temperature, a separate growth solution was poured into the seed one. The growth mixture contained 1 mL of 100 mM CTAB, 20 µL $HAuCl_4$ (20 mM), 20 µL $AgNO_3$ (5 mM) and 50 µL fresh ascorbic acid (20 mM) added at the last moment. The suspension was then heated at 70°C for 1 hour, the new chiral particles thus obtained can be conserved at room temperature for some months and washed later by centrifugation (3000 rpm, 10 min) in 1 mM CTAB.

2.4 Synthesis of chiral silver AuBP@Ag NPs (adapted from ref.[1])

In a unique vial, the following reactants were added in a row to 1 mL of 30 mM CTAC: 20 µL AuBPs diluted suspension ($[Au^0]_m^{400nm} = 0.5$ g/L), 2 µL fresh L or D-Cys (10 mM), 100 µL $AgNO_3$ (5 mM), and 100 µL fresh ascorbic acid (20 mM). The mixture was heated at 75°C for 30 minutes, the final suspension thus obtained can be stored at room temperature for a month and washed later by centrifugation (3000 rpm, 10 min) in 1 mM CTAC.

2.5 Self-assembly of chiral AuBP@Au NPs (adapted from ref.[2])

From previously synthesized chiral AuBP@Au NPs, 0.5 mL was washed three times by centrifugation (6000 rpm, 2 min) and finally redispersed in 0.25 mM CTAB. A last centrifugation was carried out and a variable amount of supernatant was replaced by pure water to reduce CTAB concentration to 0.01 mM or 0.005 mM, and set the final volume to 1 mL. After dilution, the evolution of optical properties was monitored by CD spectroscopy. Later, the diluted sample at 0.01 mM CTAB was strongly centrifuged again (10 000 rpm, 1 min) and NPs dispersed in the same solvent by sonication.

2.6 Self-assembly of chiral AuBP@Ag NPs (adapted from ref.[2])

Self-assembly of AuBP@Ag NPs was obtained by diluting a concentrated suspension of particles as described in ref.[2] . In a typical experiment, a few tens of microliters of concentrated stock solution of AuBP@Ag at 0.1 mM CTAC were introduced in a cuvette, followed by Milli-Q water to reach a final volume of 1 mL. After the mixing, the suspension was monitored by UV-Vis-NIR and CD spectroscopies. Aggregation occurred below 0.01 mM of CTAC.

2.7 Silicate encapsulation for TEM imaging (adapted from refs.[2,3])

In previous 1 mL aggregated batches of chiral NPs (30 minutes after dilution or directly after centrifugation), 1 mL of 2 mM CTAB was added to increase its concentration to around 1 mM

and inhibit momently the self-assembly progression. After, 0.5 mL of stabilized suspension was incorporated into 0.5 mL of an aqueous solution of potassium silicate (3 wt%), preheated at 60°C. The mixture was heated two more minutes at 60°C and then let cooling at RT for one hour. Finally, the silicate covered aggregates were washed three times by centrifugation (10000 rpm, 5 min) with pure water, and one drop was dried on a TEM grid for later TEM analysis and statistics.

2.8 Characterization

Transmission electron microscopy (TEM) images were captured by a Gatan ultrascan 1000 camera equipped on a JEOL 2100F operating at 200 kV. TEM statistics were performed on several dozen objects using ImageJ software. Circular dichroism (CD) and extinction spectra were recorded on a JASCO J–1700 CD spectrophotometer, using 1 cm optical glass cuvettes and pure water as reference. After, extinction dissymmetry g-factor g_{ext} of chiral systems was obtained according to the equation:

$$g_{ext} = CD/(32980 \cdot Ext) \tag{1}$$

with CD (in mdeg) the extended CD spectra recorded by the CD spectrophotometer, Ext the corresponding extinction spectra. This quantity is dimensionless and permits to get rid of concentrations in materials and becomes useful to compare quantitatively chiroptical activity through multiple chiral systems.

2.9 Additional supporting experimental data for AuBP@Au aggregates

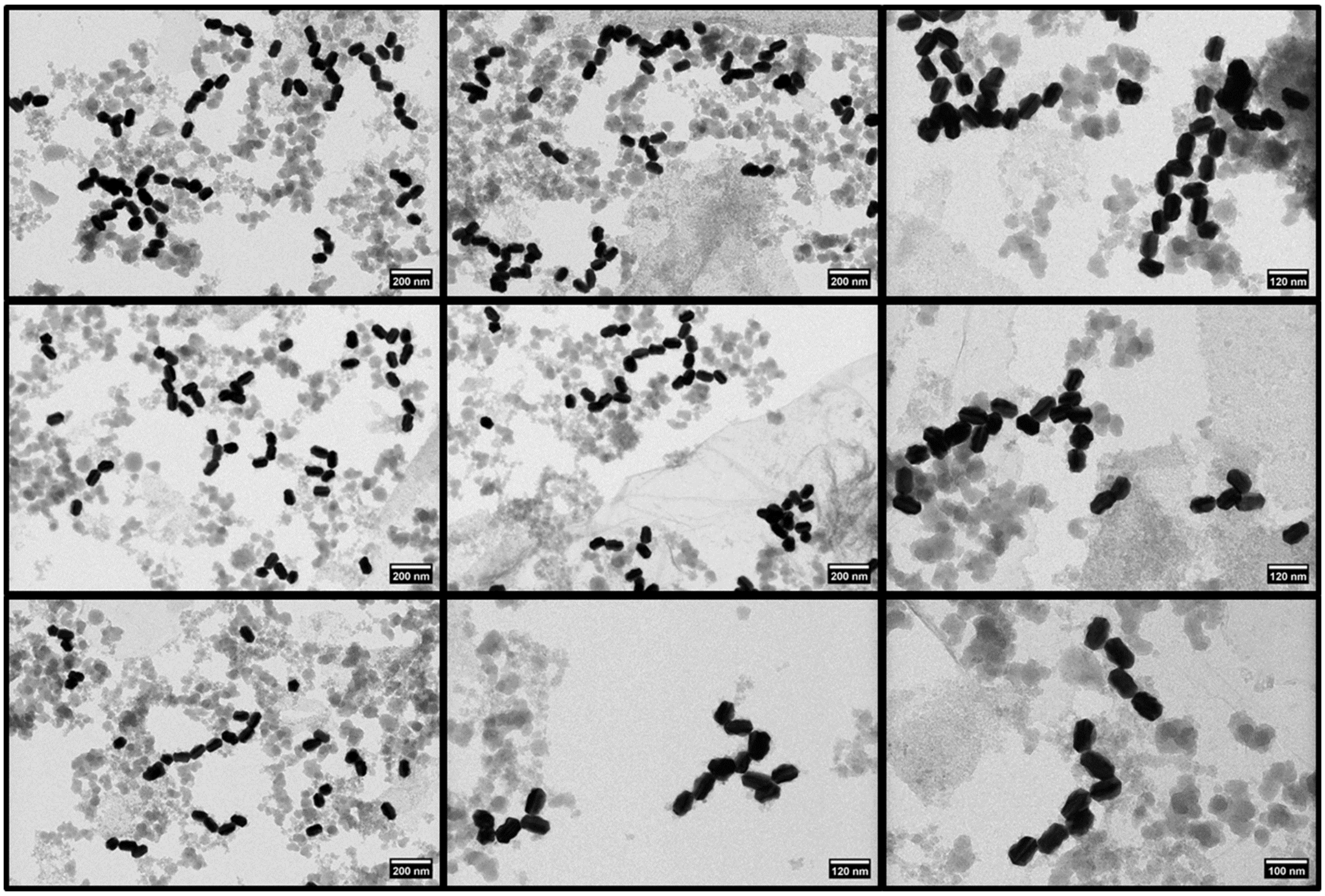

Figure S12: TEM images of diluted solution of AuBP@Au to 0.005 mM CTAB after silicate encapsulation; aggregation by dilution.

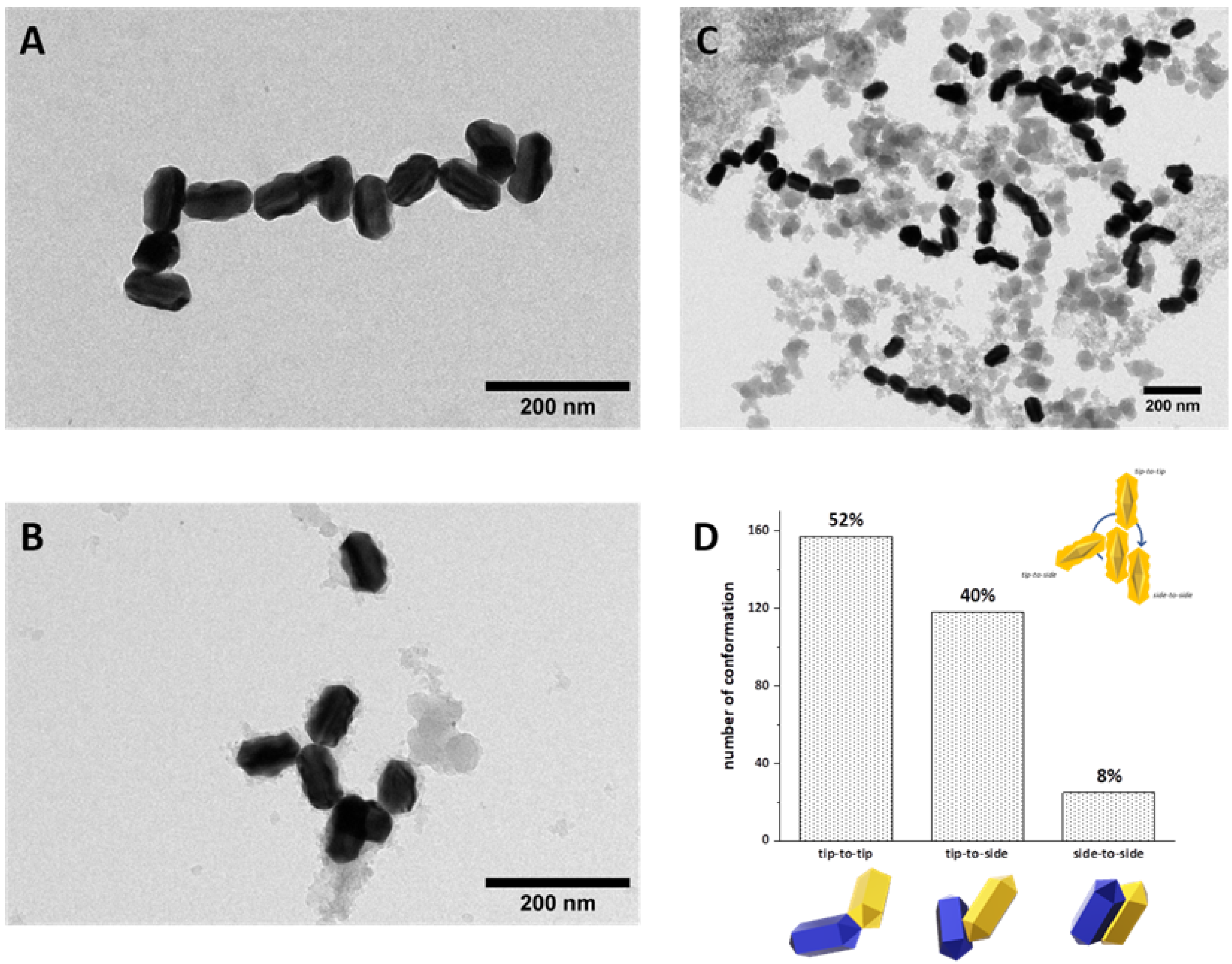

Figure S13: TEM images of centrifuged suspensions of AuBP@Au NPs at 0.01 mM CTAB (A) before and (B) after silicate encapsulation. (C) Low magnitude TEM image of diluted suspensions to 0.005 mM CTAB after silicate treatment. (D) Statistical analysis and 3D models of the three conformations possible for two pentatwinned AuBP@Au NPs assemblies (statistics over 300 close pairs of encapsulated nanoparticles on TEM images). The upper right inset shows a 2D sketch of three conformations for a dimer.

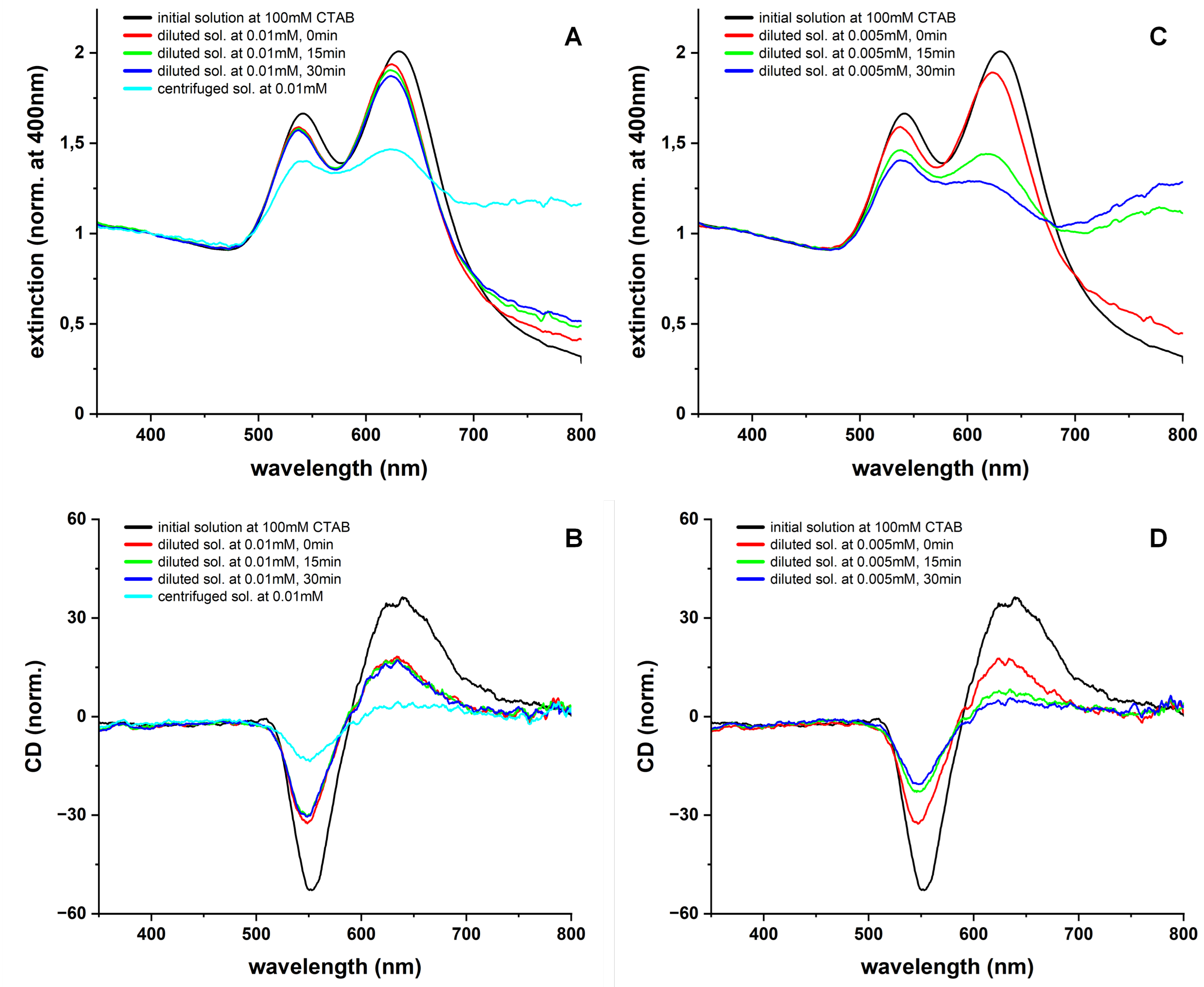

Figure S14: (A) Extinction and CD (B) spectra of diluted suspension of AuBP@Au NPs to 0.01 mM CTAB at 0, 15 and 30 minutes after water addition, then aggregated by centrifugation. (C) Extinction and CD (D) spectra of diluted suspension to 0.005 mM CTAB at 0, 15 and 30 minutes after water addition (aggregation by dilution). The original CD spectra of both diluted suspensions were multiplied by 2 to normalize NPs concentrations and compare them with initial solutions.

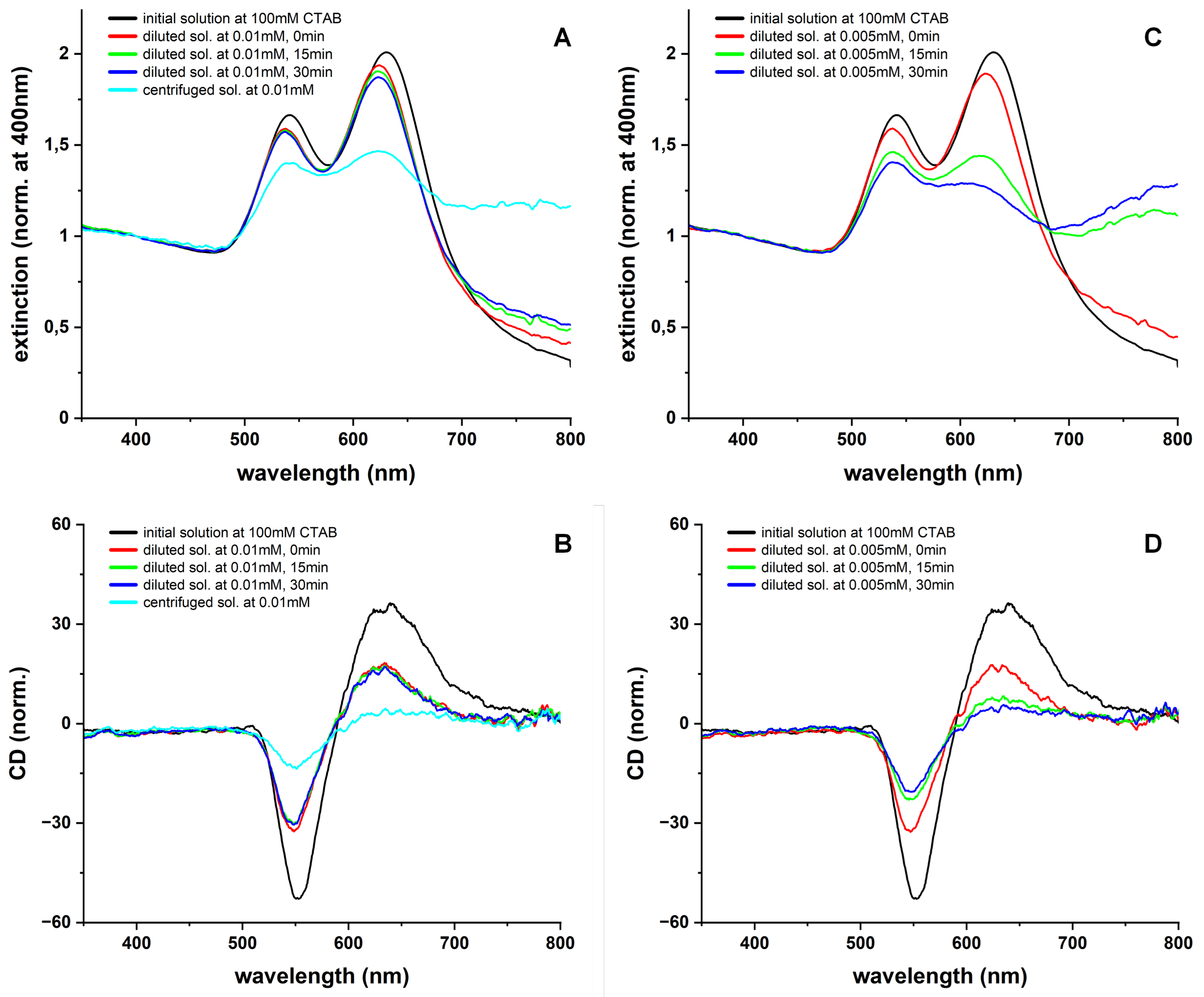

Figure S15: Intensity variation of (A) L-LSPR or T-LSPR and (B) positive or negative CD bands, for both diluted solutions at 0.01 mM and 0.005 mM CTAB at different aggregation state moments according to initial suspensions of AuBP@Au NPs.

References

(1) Carone, A.; Mariani, P.; Désert, A.; Romanelli, M.; Marcheselli, J.; Garavelli, M.; Corni, S.; Rivalta, I.; Parola, S. Insight on Chirality Encoding from Small Thiolated Molecule to Plasmonic Au@Ag and Au@Au Nanoparticles. *ACS Nano* **2022**, *16*, 1089–1101.

(2) Carone, A.; Mariani, P.; Désert, A.; Parola, S. Colloidal Assemblies of Chiral Plasmonic Nanoparticles Induce Tunable Circular Dichroism Response. *Adv. Opt. Mater.* **2023**, *11*, 2300119.

(3) Carone, A.; Emilsson, S.; Mariani, P.; Désert, A.; Parola, S. Gold nanoparticle shape dependence of colloidal stability domains. *Nanoscale Adv.* **2023**, *5*, 2017–2026.

(4) Hohenester, U.; Trügler, A. MNPBEM–A Matlab toolbox for the simulation of plasmonic nanoparticles. *Comput. Phys. Commun.* **2012**, *183*, 370–381.

(5) Johnson, P. B.; Christy, R.-W. Optical constants of the noble metals. *Phys. Rev. B* **1972**, *6*, 4370.

(6) Marcheselli, J.; Chateau, D.; Lerouge, F.; Baldeck, P.; Andraud, C.; Parola, S.; Baroni, S.; Corni, S.; Garavelli, M.; Rivalta, I. Simulating plasmon resonances of gold nanoparticles with bipyramidal shapes by boundary element methods. *J. Chem. Theory Comput.* **2020**, *16*, 3807–3815.

(7) Chateau, D.; Desert, A.; Lerouge, F.; Landaburu, G.; Santucci, S.; Parola, S. Beyond the Concentration Limitation in the Synthesis of Nanobipyramids and Other Pentatwinned Gold Nanostructures. *ACS Appl. Mater. Interfaces* **2019**, *11*, 39068–39076.
